# Ovarian cancer stem cells and macrophages reciprocally interact through the WNT pathway to promote pro-tumoral and malignant phenotypes in 3D engineered microenvironments

**DOI:** 10.1186/s40425-019-0666-1

**Published:** 2019-07-19

**Authors:** Shreya Raghavan, Pooja Mehta, Yuying Xie, Yu L. Lei, Geeta Mehta

**Affiliations:** 1 Department of Materials Science and Engineering, 2800 Plymouth Rd, Building 28, Room 3044W, Ann Arbor, MI 48109 USA; 2 Department of Biomedical Engineering, 2800 Plymouth Rd, Building 28, Room 3044W, Ann Arbor, MI 48109 USA; 3 Department of Macromolecular Sciences and Engineering, 2800 Plymouth Rd, Building 28, Room 3044W, Ann Arbor, MI 48109 USA; 4 Department of Periodontics and Oral Medicine and Department of Otolaryngology Head and Neck Surgery, Ann Arbor, USA; 5 Rogel Cancer Center, North Campus Research Complex, University of Michigan, 2800 Plymouth Rd, Building 28, Room 3044W, Ann Arbor, MI 48109 USA; 6 Department of Computational Mathematics, Science, and Engineering, Michigan State University, East Lansing, MI 48823 USA

## Abstract

**Background:**

Innate immune cells such as macrophages are abundantly present within malignant ascites, where they share the microenvironment with ovarian cancer stem cells (CSC).

**Methods:**

To mimic this malignant ascites microenvironment, we created a hanging-drop hetero-spheroid model to bring CSCs and macrophages in close association. Within these hetero-spheroids, CD68^+^ macrophages (derived from U937 or peripheral blood monocytes) make up ~ 20% of the population, while the rest are ovarian cancer cells and ovarian cancer stem cells (derived from the high grade serous ovarian cancer cell line, OVCAR3).

**Results:**

Our results indicate that CSCs drive the upregulation of M2 macrophage marker CD206 within hetero-spheroids, compared to bulk ovarian cancer cells, implying an inherently more immuno-suppressive program. Moreover, an increased maintenance of elevated aldehyde dehydrogenase (ALDH) activity is noted within hetero-spheroids that include pre-polarized CD206^+^ M2 macrophages, implying a reciprocal interaction that drives pro-tumoral activation as well as CSC self-renewal. Consistent with enriched CSCs, we also observe increased levels of pro-tumoral IL-10 and IL-6 cytokines in the CSC/M2-macrophage hetero-spheroids. CSC/M2-macrophage hetero-spheroids are also less sensitive to the chemotherapeutic agent carboplatin and are subsequently more invasive in transwell assays. Using inhibitors of WNT secretion in both CSCs and macrophages, we found that CSC-derived WNT ligands drove CD206^+^ M2 macrophage activation, and that, conversely, macrophage-derived WNT ligands enriched ALDH^+^ cells within the CSC compartment of hetero-spheroids. Upon examination of specific WNT ligand expression within the monocyte-derived macrophage system, we observed a significant elevation in gene expression for *WNT5B*. In CSCs co-cultured with macrophages within hetero-spheroids, increases in several WNT ligands were observed, and this increase was significantly inhibited when WNT5B was knocked down in macrophages.

**Conclusions:**

Our data implies that macrophage- initiated WNT signaling could play a significant role in the maintenance of stemness, and the resulting phenotypes of chemoresistance and invasiveness. Our results indicate paracrine WNT activation during CSC/M2 macrophages interaction constitutes a positive feedback loop that likely contributes to the more aggressive phenotype, which makes the WNT pathway a potential target to reduce the CSC and M2 macrophage compartments in the tumor microenvironment.

**Electronic supplementary material:**

The online version of this article (10.1186/s40425-019-0666-1) contains supplementary material, which is available to authorized users.

## Introduction

Late stage epithelial ovarian cancer presents frequently with peritoneal carcinomatosis, and is associated with the formation of malignant ascites. Exfoliated ovarian cancer cells exist as spheroids within the ascites microenvironment, and there is also an enrichment of ovarian cancer stem cells (CSCs) within the peritoneal fluid [1–3]. Within the malignant ascites, CSCs interact with a variety of host cells including different immune subsets in the presence of a complex cytokine/chemokine network, ultimately leading to trans-coelomic metastasis [4–6]. Also found in abundance within the peritoneal ascites fluid are macrophages, which are generally believed to be polarized and educated by tumor-derived factors into an M2-resembling pro-tumoral phenotype. In fact, ovarian cancer cells interact with macrophages in anchorage independent conditions and grow as spheroids within the malignant ascites, which can result in tumor metastasis even during the early stage of peritoneal dissemination [7].

In the ovarian cancer microenvironment, macrophages are activated into a tumor-associated macrophage (M2-like, “M2”, alternatively activated) phenotype primarily through education by tumor-derived cytokines, chemokines and other tumor cell-derived factors [8, 9]. Tumor-associated macrophages within malignant ascites have a bipolar expression spectrum, ranging from M1-like to M2-like phenotypes. However, M2-like macrophages are the primary pro-tumoral phenotype in the peritoneal cavity. In fact, a high ratio of M1/M2 macrophages is associated with an improved prognosis in ovarian cancer, whereas lower M1/M2 ratio is indicative of a poor prognosis [10–12].

Little is known about the specific role of cancer *stem cells* in macrophage polarization and activation. Even less is known about the reciprocal interactions between CSCs and macrophages. Given the enriched presence of macrophages and CSCs within the malignant ascites, their interaction may be critical for regulating the progression and drug response of ovarian cancer. Therefore, in this study, we used a previously established in vitro hanging drop spheroid model [13–15] to dissect the reciprocal interactions between the CSCs in tumor spheroids and macrophages. The hanging drop spheroid model allows the formation of stable spheroids in a non-adherent 3D in vitro environment, similar to the aggregation of ovarian cancer cells floating within the malignant ascites in anchorage-independent conditions. We previously demonstrated that the hanging drop spheroid model maintains CSCs derived from primary patient samples with high fidelity, and preserves responses to chemotherapeutic agents similar to mouse xenograft models [13].

The importance and abundance of WNT-signaling have been demonstrated in ovarian development, tumorigenesis and stem cell maintenance [16, 17]. In the tumor immune microenvironment, activated WNT/β-catenin signaling can suppress the recruitment of dendritic cells, thereby limiting T-cell priming, and intra-tumoral T-cell accumulation [18]. WNT signaling is also heavily involved in the activation of macrophages [19, 20]. Importantly, paracrine WNT signaling loops between M2-like macrophages and tumor cells contribute to tumorigenesis and invasiveness [21, 22].

We hypothesized that any trophic interactions between CSCs and macrophages may involve a WNT-dependent pathway. Therefore, using the hanging drop spheroid model, we sought to understand pro-tumoral macrophage activation in response to CSCs, and changes in the CSC compartment itself in response to activated macrophages. We evaluated the WNT pathway in CSC-macrophage interactions, and whether that corresponded to functional changes in chemoresistance or invasion of CSC spheroids. Insight into WNT involvement in CSC-macrophage interactions could provide new targetable avenues to reduce CSC-burden in ovarian cancer, thereby limiting metastatic and recurrent disease.

## Materials and methods

### Materials

Cell lines were purchased from ATCC (Manassas, VA). Peripheral blood mononuclear cells (PBMCs) were purified from buffy coats from healthy donors through Ficoll-Paque gradient centrifugation. Cytokines were purchased from Peprotech Inc., and all other tissue culture supplements from Life Technologies, and chemicals from Sigma Aldrich (St. Louis, MO) unless otherwise specified. Compounds Ruxolitinib and sc144 were a generous gift from the laboratory of Dr. Karen McLean. Viral vectors were purchased from Sigma Aldrich and packaged at the University of Michigan Viral Vector core.

### Derivation and polarization of macrophages from U937 cell line, and PBMCs

U937 cells were cultured in suspension in RPMI supplemented with 10% heat-inactivated fetal calf serum (Atlanta Biologics) and 1x antibiotics/antimycotics. Cells were harvested, and suspended at 2500cells/ml and treated with 5 ng/ml phorbol myristate acetate (PMA). 20 μl of this suspension was plated onto each well of a hanging drop array plate, to allow monocytes to differentiate into macrophages in suspension culture. For PBMCs, cells were plated onto tissue culture dishes, and the non-adherent cell fraction was discarded following 24 h of attachment. PBMCs were then detached from the plate, and plated onto hanging drop arrays at 500 cells/drop. At the end of 24 h, each well was left untreated to derive M0 resting macrophages, or treated with 20 ng/ml recombinant human M-CSF and 20 ng/ml IL-4 to derive activated M2-like macrophages for the next 48 h. For brevity, figure captions refer to IL-4/MCSF activated macrophages as M2, to indicate the M2-like, alternatively activated phenotype. Macrophages were harvested from hanging drops and assessed for differentiation and polarization using flow analysis, described below. Harvested macrophage aggregates were also subjected to subsequent qPCR analysis, or used to manufacture hetero-spheroids.

### Isolation of ovarian CSCs from ovarian cancer cell lines

Ovarian CSCs were isolated from the serous ovarian cancer cell lines OVCAR3 (used under passage 35) as described previously [13]. Briefly, cells were harvested and incubated with ALDEFLUOR reagent, and CD133 antibody, and sorted using flow cytometry for cells positive for elevated ALDH and CD133 positivity. Appropriate DEAB and isotype controls were used for both assays, to determine gate settings as described previously. CSCs were freshly sorted and used to make hetero-spheroids < 24 h after flow sorting.

### Formation of mono- and hetero-spheroids from CSCs and macrophages

Spheroids were generated on a hanging drop array plate from CSCs and macrophages adapting protocols described previously [13–15]. For mono-spheroids, 100 CSCs or the unsorted bulk of OVCAR3 cells were seeded per hanging drop and allowed to form spheroids. Macrophages were harvested from hanging drops following the differentiation protocol described in Section 2.2. Macrophages and CSCs were combined and plated onto hanging drop arrays such that each drop contained 100 CSCs and 100 M0/M2 macrophages. Mono-spheroids contained 100 CSCs or bulk unsorted OVCAR3 cells. Following 4–5 days in hanging drop array culture, spheroid formation was tracked using live cell microscopy and routinely fed to maintain a ~ 20 μl drop volume. Spheroids were used for subsequent flow analysis, qPCR or lysed to obtain protein for immunoblots. In some instances, hetero-spheroids were manufactured from CSCs stably expressing GFP, and fluorescent activated cell sorting was utilized to separate the GFP^+^ CSC compartment from hetero-spheroids for further analysis.

### Flow cytometry analysis

Flow cytometry analysis was performed following protocols established in our lab previously [13]. CD68-APC (Miltenyi Biotech, Germany) antibody was used, with its associated APC-isotype control, to identify CD68^+^ macrophages, in differentiated U937 or PBMC monocytes, and hetero-spheroids. For CD68 flow analysis, samples were fixed in methanol at − 20 °C, for 1 h, followed by a PBS wash to remove methanol, re-suspension in FACS Buffer (PBS + 2% FBS) before antibody incubation.

Two kinds of flow cytometry based experiments were carried out to characterize hetero-spheroids: i) macrophage polarization was assessed by CD68^+^ and CD206^+^ using flow cytometry; ii) Stemness was assessed by ALDEFLUOR assay for observation of elevated ALDH activity using flow cytometry, using protocols established previously [13]. Briefly, hetero-spheroids were harvested in FACS buffer, and triturated to single-cell suspensions. Appropriate isotype controls were used for conjugated antibodies, to set gates to observe CD68 and CD206. For ALDH, a molar excess of the DEAB inhibitor was used to determine positive gates per the manufacturer’s protocol, to identify elevated ALDH activity. ALDH activity was assessed following 48-h treatment with the JAK1/2 inhibitor, Ruxolitinib, or the GP130 inhibitor, sc144, or the human anti IL-6 antibody, Tocilizumab (Actemra, Genentech). Flow cytometry was performed on the Attune acoustic focusing flow cytometer (Applied Biosystems). Flow sorting was performed on the Astrios (Beckman Coulter).

### Gene expression via qPCR

RNA was extracted from harvested macrophages or hetero-spheroids using the RNeasy extraction kit (Qiagen). Extracted RNA was assessed for concentration and purity using a Nanodrop 2000 (Thermo Fisher Scientific) spectrophotometer. RNA was transcribed to cDNA using the High-fidelity cDNA Transcription kit (Life Technologies), and qPCR was carried out in the 96-well format using the 7900HT platform (Applied Biosystems). For macrophage polarization, *CD163* and *CD206* were assessed*. IL-10* was additionally assessed in hetero-spheroids. Lastly, *WNT* ligands were also assessed in hetero-spheroids. Gene expression differences were quantified using the 2ΔΔC_T_ method, using GAPDH as the housekeeping control, and reported as fold change compared to a control sample. For macrophages, controls were undifferentiated monocytes. For hetero-spheroids, control samples were bulk OVCAR3 spheroids. qPCR experiments were run in triplicates, with 2–3 independent samples. A list of primers used in the qPCR experiments is provided in Additional file 1: Table S1.

### Quantification of cytokines using ELISA

For ELISA assays, media was harvested from 50 spheroids (macrophages, OVCAR3, CSC, CSC/M2 or CSC/sh-WNT5B M2). ELISA assays were performed on the Duoset ELISA system (R&D Biosystems, Minneapolis MN) following the manufacturer’s protocol, modified to include an overnight sample incubation. Cytokines analyzed included IL-10 and IL-6. Standard curves were generated for each cytokine, and analyte concentration was assessed using a four parametric ELISA curve, to determine the amount of IL-10 or IL-6 released. ELISA assays and data analysis were performed at the Immunological Monitoring Core at the Rogel Cancer Center, University of Michigan.

### Assessment of chemoresistance in hetero-spheroids

For chemoresistance, hetero-spheroids were treated with carboplatin, to a final concentration of 300 μM, within 20 μl drops, for 48 h. At the end of 48 h, the MTS reagent (Abcam) was added to drops at a 1/10 dilution, and allowed to incubate at 37 °C for 2.5 h. At the end of the incubation period, absorbance was read on the hetero-spheroids at 590 nm, according to manufacturer’s protocols. Untreated hetero-spheroids were used as controls, to normalize the absorbance to identify the effect of drug treatment on cellular viability. Results were quantified as normalized cell viability, based on untreated controls. Experiments were repeated with 3–5 biological replicates for statistical analysis.

### Assessment of migration of hetero-spheroids

In order to quantify invasiveness of hetero-spheroids, 8 μm transwell inserts were placed in each well of a 24 well plate. 10 CSC mono-spheroids or CSC/M2, CSC/scramble M2, CSC/sh-WNT5B M2 hetero-spheroids were harvested at Day 5 from hanging drop arrays, and placed on the top chamber of a transwell insert. The bottom chamber was filled with 400 μl of fresh medium, so only the bottom of the transwell insert was immersed in medium. Following 3 days, the transwell insert was removed, and several images of the bottom of the 24 well were obtained using phase contrast microscopy. Image J was used to quantify the number of cells in a field of view. At least four random non-overlapping fields of view were counted from each experiment, to find the number of cells that migrated through the transwell insert to the bottom of the well.

### Immuno-blotting for β-catenin

Hetero-spheroids were harvested and lysed in 200 μl of Radio-immunoprecipitation assay (RIPA) Buffer, sonicated for 30s on ice with a probe sonicator. Extracted concentration was measured using the BCA Assay Reagent (Pierce) following manufacturer’s protocol for a 96-well format. Subsequently, 50 μg of protein from each sample was loaded onto 4–20% gradient polyacrylamide gels (Biorad), and separated electrophoretically, transferred to a PVDF membrane. Transferred membranes were blocked with 5% non-fat milk, and probed with β-catenin (R&D Biosystems) overnight at 4 °C, washed with TBST buffer, and probed with an appropriate HRP-conjugated secondary antibody. β-Actin was used as a loading control to determine changes in β-catenin expression among samples. ECL reagent (Pierce Protein Biology) was used to visualize bands in a Biorad ChemiDoc Touch instrument. Digital images acquired were processed through NIH Image J, and band analysis tools were used for densitometry. Band densities were normalized against the loading control β-Actin, to determine changes.

### Knock-down of WNT5B in macrophages

Mission shRNA plasmids were obtained transformed into *E. coli* from Sigma Aldrich, targeted to WNT5B (TRCN0000123194). Transformed *E. coli* were grown in LB medium. Plasmid DNA was isolated using the Promega DNA isolation kit following manufacturer’s protocols, and 2.5 μg of DNA was transfected along with packaging pro-viral plasmids into HEK293-T cells. Lentivirus particles were isolated at 1X concentration by the University of Michigan Viral Vector Core. 1 × 10^5^ cells were transduced with 3 μg/ml polybrene and 0.5X lentivirus for 30 min at 800 g, at 32 °C in a centrifuge. Resulting pellets were re-suspended in fully supplemented growth medium for 72 h. At the end of 72 h, cells were harvested for qPCR analysis, or for macrophage differentiation and further experimentation. Lentiviruses were packaged to express shRNA targeting WNT5B (sh-WNT5B), or scrambled shRNA (sh-scramble). Sh-WNT5B or sh-scramble treated U937 monocytes were differentiated and activated into macrophages following protocols described in section 2.2.

### Assessment of tumorigenicity in vivo of hetero-spheroids

CSC mono-spheroids and CSC/M2 and CSC/sh-WNT5B M2 hetero-spheroids were generated following protocols outlined in Section 2.4. CSCs were GFP tagged in these spheroids, and after five days of hetero-spheroid culture, CSCs were isolated using the GFP label prior to sub-cutaneous injection into NSG mice. Each tumor received CSCs from 10 spheroids. Tumor initiation and monitoring protocols were performed as described previously [13]. When palpable tumors were observed, tocilizumab (10 mg/kg, intra peritoneally) treatment began 3 times/week. Tumors were allowed to grow till the end-point was reached for maximum tumor burden, and mice were euthanized. Tumors were dissected, and routine paraffin histology and H&E staining was performed, to understand any changes in histology. RNA was isolated from tumors following protocols outlined in Section 2.6, and subject to qPCR for *ALDH1A1,* and several other Wnt ligands.

### Data analysis and statistics

Experiments were carried out using 3–5 biological replicates for U937-derived macrophages, and OVCAR3-derived CSCs. GraphPad Prism 5.0 (www.graphpad.com) was utilized to perform all statistical analysis. When appropriate, one-way ANOVAs were used to test significant differences, and if differences were observed, indicated with symbols and a significance level.

## Results

### Monocyte-derived macrophages can be differentiated and activated in 3D hanging drop cultures

**Fig. 1:**
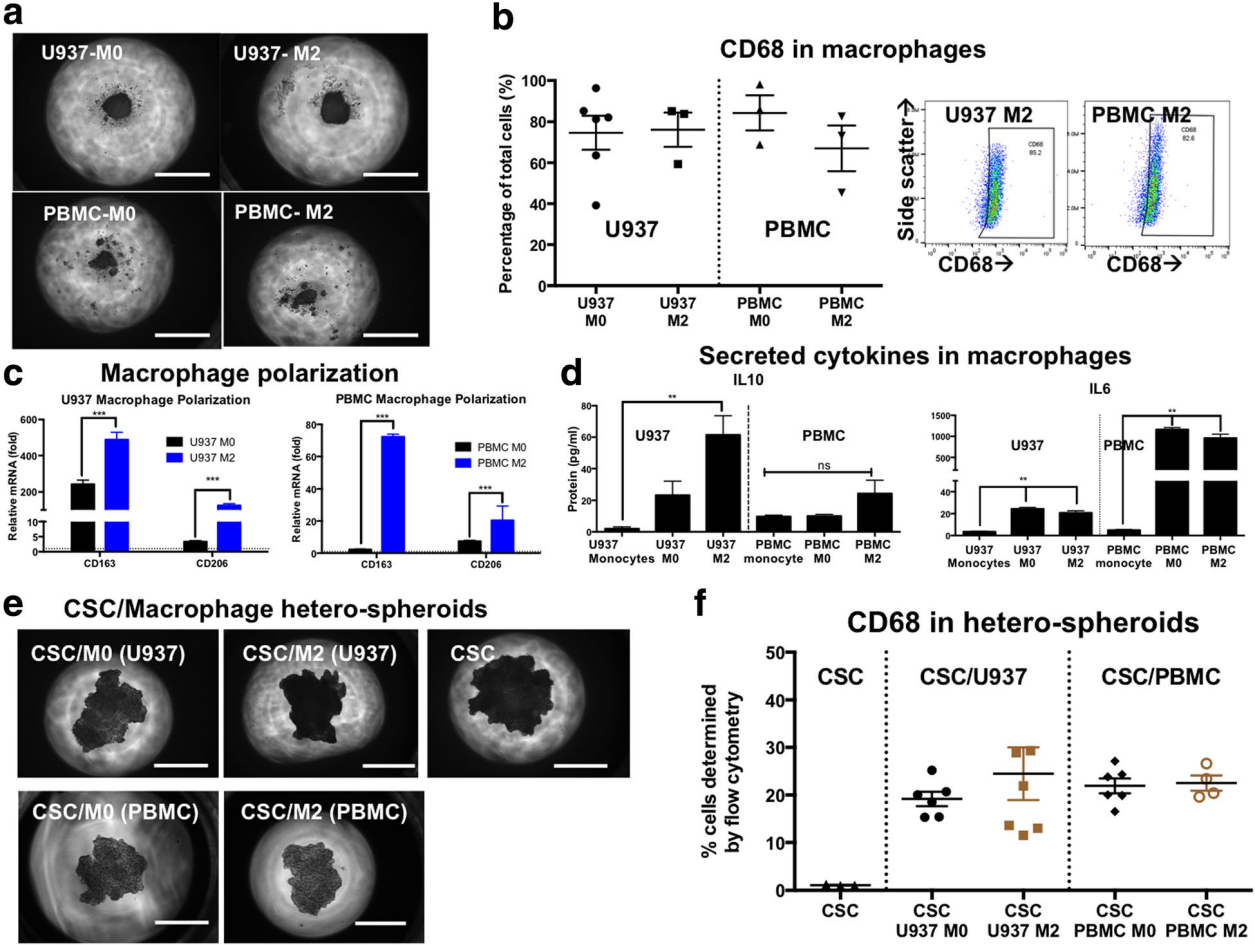
Hetero-spheroids derived from monocyte-derived macrophages and ovarian cancer stem cells. **a** Monocytes from the U937 cell line or peripheral blood monocytes (PBMC) were plated into hanging drop arrays, and differentiated to M0 macrophages with phorbol ester treatment, or activated with IL-4 and MCSF treatment. Differentiated and activated macrophages formed compact spheroid-like aggregates. **b** Differentiated M0 and M2 macrophages all expressed pan macrophage marker, CD68 indicating 75–80% differentiation efficiency from monocytes to macrophages. CD68 expression was evaluated using flow cytometry analysis, with representative plots. **c** Polarization was assessed using qPCR analysis for two M2 genes, CD163 and CD206. Both U937 and PBMC macrophages expressed significantly higher levels of CD163 and CD206 genes, compared to untreated undifferentiated monocytes. **d** M2 differentiated macrophages secreted higher amounts of the immuno-suppressive cytokine IL-10, and the pro-tumoral cytokine IL-6. **e** CSCs were derived from the OVCAR3 cell line based on ALDH^+^ CD133^+^ expression. Hetero-spheroids were generated using differentiated U937 or PBMC M0 macrophages and CSCs or activated U937 or PBMC M2 macrophages and CSCs. Representative phase contrast images of hetero-spheroids seen at Day 5 following formation indicate compact spheroids, similar in size to CSC mono-spheroids generated from the same number of CSCs/spheroid. **f** Hetero-spheroids retain ~ 20% CD68 expression, indicating that at day 5, CD68^+^ macrophages constitute 20% of the population of cells. Scale bar = 200 μm

The monocytic cell line, U937, or healthy-donor derived PBMCs were placed in hanging drop cultures. In the presence of PMA, U937 and PBMC monocytes differentiated into macrophages, and over the course of 72 h, they were organized as a compacted mass of cells within hanging drop cultures (Fig. 1a). Monocytes with no PMA stimulation, in contrast, were extremely loosely aggregated and did not form compact spheroids. Differentiated macrophages were indicated as M0 macrophages, implying differentiation with no cytokine stimulation. 74.6 ± 8.2% of U937 monocytes differentiated into M0 macrophages expressed the pan macrophage marker CD68. Similarly, PBMCs also differentiated into macrophages upon PMA stimulation, with 84.3 ± 8.5% CD68 expression (Fig. 1b). Monocytes were also differentiated and polarized into an alternatively activated phenotype (M2; Fig. 1a). These macrophages were derived with PMA stimulation in the presence of IL-4 and M-CSF. Alternately activated M2-like macrophages, either from U937 or PBMCs had an associated increase in gene expression of CD163 and CD206 (Fig. 1c). Immuno-suppressive cytokine IL-10 and tumor-promoting cytokine IL-6 were elevated in M2-polarized macrophages from both U937 and PBMC compared to M0 macrophages, or undifferentiated monocytes, indicating a shift in phenotype between M0 and M2-like macrophages derived using this culture system (Fig. 1d). In summary, macrophages could be derived from U937 or PBMC monocytes using the 3D hanging drop culture system, and could be further activated into a M2-phenotype. 3D differentiation and activation was similar in terms of gene expression to conventionally activated macrophages in 2D culture systems, including elevated arginase enzyme activity (Additional file 1: Figure S1).

### Hetero-spheroids can be derived from ovarian cancer stem cells and macrophages using hanging drop cultures

Hetero-spheroids combining ovarian CSCs derived from the OVCAR3 cell line and monocyte-derived macrophages (U937 or PBMC) in a starting ratio of 1:1 were generated using hanging drop arrays. Phase contrast images of hetero-spheroids at Day 5 indicate compact spheroid formation with tight defined boundaries (Fig. 1e). No significant differences in size or proliferative index were observed between CSC mono-spheroids, and CSC/M0 or CSC/M2 hetero-spheroids Additional file 1: Figure S2). Hetero-spheroids retained between 19.2 ± 1.5%-26.3 ± 1.8% expression of CD68^+^ macrophages at Day 5 (Fig. 1f). Gating strategy for flow analysis is presented in Additional file 1: Figure S3. CSC/M0 or CSC/M2 hetero-spheroids thereby maintained robust macrophage populations within the spheroids, while maintaining compact spheroid architecture for 5 days.

### Ovarian cancer stem cells drive CD206 expression in M0 monocyte-derived macrophages within hetero-spheroids through IL10 and Wnt-signaling

**Fig. 2:**
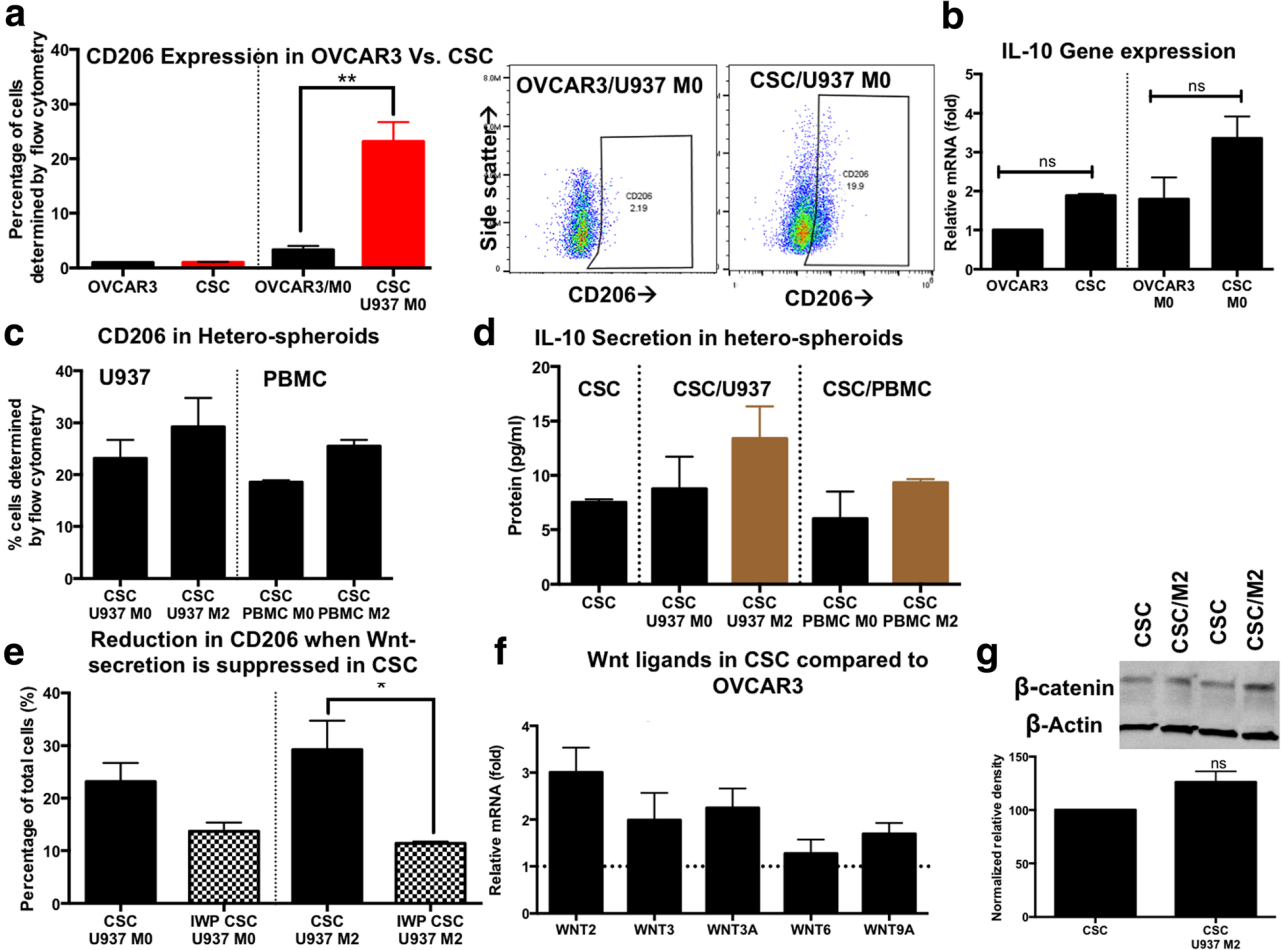
Hetero-spheroids drive CD206 polarization in monocyte-derived macrophages. **a** Hetero-spheroids were generated from bulk unsorted OVCAR3 cells and M0 macrophages (OVCAR3/U937 M0), and CSC/U937 M0. Flow analysis for the macrophage polarization marker CD206 indicated that while OVCAR3 and CSCs by themselves do not express CD206, CSCs drive CD206 expression in previously CD206^−^ M0 macrophages within hetero-spheroids at significantly (**p < 0.001, one-way ANOVA) higher levels than OVCAR3. Representative flow analysis plots are shown. **b** *IL10* gene expression was evaluated using qPCR in mono- and hetero-spheroids, indicating that CSCs had a 2-fold increased gene expression of *IL10*, likely driving CD206 expression in M0 macrophages to higher extents than OVCAR3. **c** CD206 expression was maintained at elevated levels (18.5–29.24%) in all CSC hetero-spheroid conditions, indicating that the activated M2 phenotype was maintained in macrophages within hetero-spheroids. **d** Levels of secreted IL-10 were mildly elevated in CSC/M2 hetero-spheroids, but were also similar across all CSC/macrophage hetero-spheroids, indicating the presence of the immuno-suppressive cytokine. **e** When CSCs were pre-treated with the Wnt secretion inhibitor, IWP-2, significantly lower (*p < 0.05,one-way ANOVA) CD206 expression was observed in CSC/M2 hetero-spheroids, implying the importance of CSC-derived Wnt ligands in the maintenance of M2 activation in macrophages. **f** qPCR analysis revealed that gene expression of several Wnt ligands were elevated in CSC compared to bulk OVCAR3 spheroids (dotted line = no change); (**g**) Densitometric measurements of immunoblots for β-catenin compared to the loading control β-actin indicated that CSC/M2 spheroids had a 26% increase in β-catenin expression, compared to CSC mono-spheroids, indicating higher Wnt/β-catenin activity in CSC/M2 hetero-spheroids

In order to understand if there are differences in bulk ovarian cancer cells (OVCAR3), and ovarian cancer stem cells (ALDH^+^ CD133^+^ CSC) in their ability to drive an immuno-suppressive macrophage phenotype, we generated hetero-spheroids from OVCAR3/M0 and CSC/M0. OVCAR3 or CSC mono-spheroids demonstrate minimal expression of CD206 (0.9–1%, Fig. 2a, Additional file 1: Figure S4). When comparing differences in CD206 expression between OVCAR3/M0 or CSC/M0 hetero-spheroids, a significant (**p < 0.001, one-way ANOVA) 20% increase in CD206 was observed with CSC co-culture (Fig. 2a), indicating that ovarian CSCs drive an immuno-suppressive phenotype in macrophages compared to bulk ovarian cancer cells. CSC/M2 hetero-spheroids also expressed another alternately activated M2 macrophage marker, CD163 (Additional file 1: Figure S5). We sought to explore whether there are differences in the immuno-suppressive cytokine IL-10 between OVCAR3 and CSC spheroids. We found that IL-10 gene expression was significantly elevated (2-fold) in CSC mono-spheroids compared to OVCAR3, which was even more pronounced in CSC/M0 spheroids compared to OVCAR3/M0 (Fig. 2b). In hetero-spheroids derived from M2 macrophages and CSCs, CD206 expression was maintained through co-culture (Fig. 2c). Higher secreted IL-10 levels were observed in CSC/M2 hetero-spheroids (Fig. 2d).

We then explored the possibility of a factor other than IL-10 driving CD206 expression in macrophages. Given the importance of the WNT signaling cascade in both Ovarian CSC maintenance as well as macrophage polarization and activation, we inhibited all WNT secretion from Ovarian CSCs using an inhibitor, IWP-2. When hetero-spheroids were generated from IWP-2 treated CSCs and M0 or M2 macrophages, CD206 expression significantly decreased (44–62%) in hetero-spheroids (*p < 0.05, one-way ANOVA; Fig. 2e), with no associated change in CD68 expression. Concomitantly, we found the gene expression of several Wnt ligands elevated close to 2-fold in CSC compared to bulk OVCAR3 spheroids (Fig. 2f). Furthermore, we found an associated 30% increase in beta-catenin protein expression, indicating an increased canonical Wnt signaling axis in CSC/M2 spheroids (Fig. 2g).

In short, in CSC-macrophage interactions within hetero-spheroids, we found not only elevated levels of the immuno-suppressive cytokine IL-10, but also of several WNT ligands. We also noted a dependency of M2 macrophage activation on CSC-derived WNT ligands.

### Alternatively activated M2 macrophages increase ovarian CSC populations within hetero-spheroids through IL-6 signaling, and are more chemoresistant and invasive

**Fig. 3:**
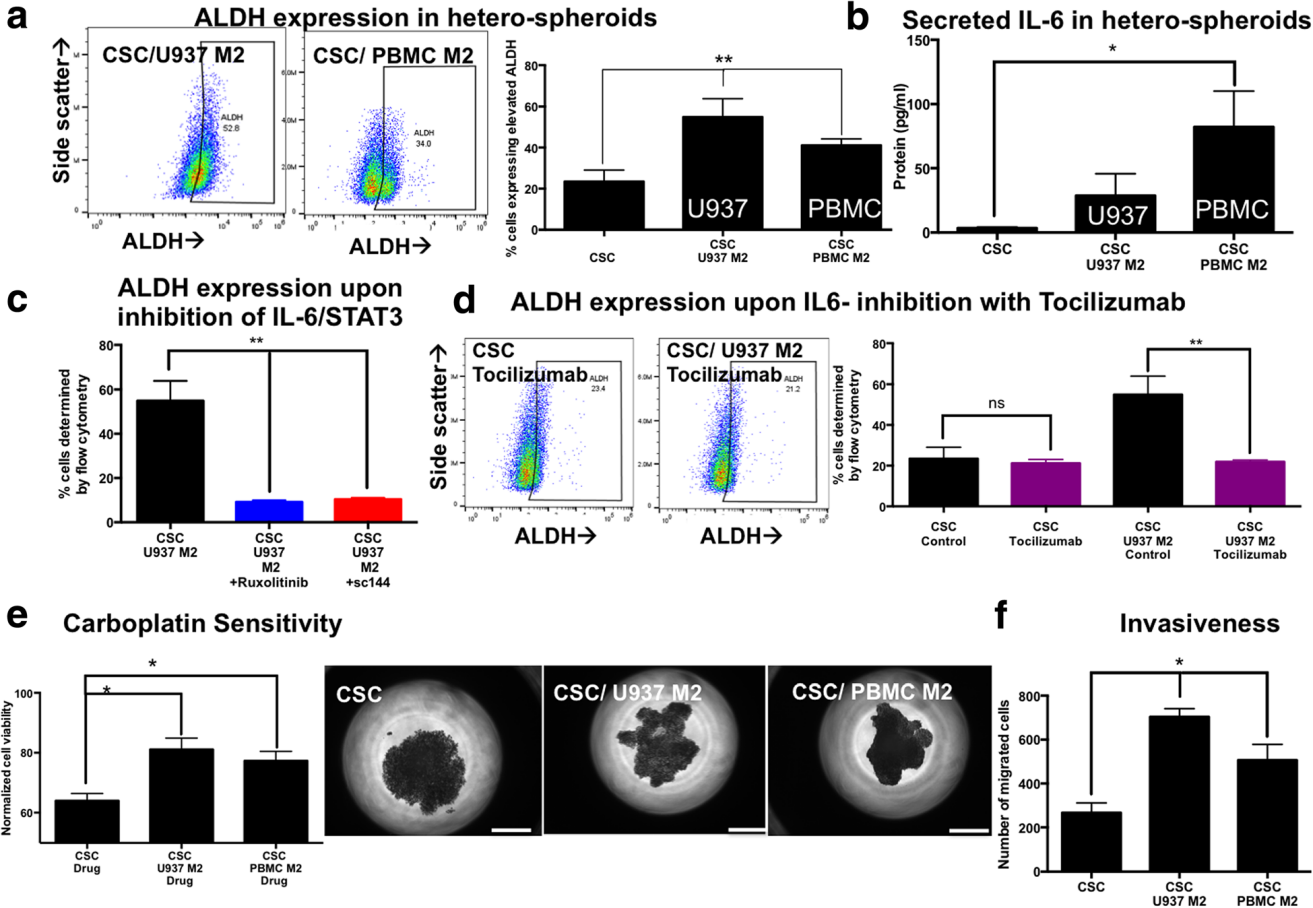
M2-polarized macrophages increase ALDH+ in CSC/M2 hetero-spheroids. **a** Elevated ALDH activity was assessed using the ALDEFLUOR flow cytometric analysis assay, with representative flow plots demonstrated. CSC/M2 hetero-spheroids, whether U937 or PBMC, have significantly (**p < 0.001, one-way ANOVA) elevated ALDH^+^ expression (1.8–2.4 fold). **b** Secreted pro-tumoral cytokine IL6 is elevated in hetero-spheroids compared to CSC mono-spheroids. **c** We suppressed signaling through the IL6/STAT3 axis using two inhibitors Ruxolitinib and sc144 in hetero-spheroids, and observed that this suppression also significantly (**p < 0.001, one-way ANOVA) decreased the ALDH^+^ enrichment within CSC/M2 hetero-spheroids. **d** Concomitant with the ALDH expression within hetero-spheroids, sensitivity to carboplatin was evaluated using the MTS assay. Normalized absorbance values indicated that CSC/M2 hetero-spheroids were significantly (*p < 0.05, one-way ANOVA) more resistant to carboplatin treatment compared to CSC mono-spheroids made with the same number of CSCs. **e** Representative phase contrast images indicate the loss of the tight spheroid boundaries in the CSC mono-spheroids in response to carboplatin treatment, indicative of cell death. The extent of the loss of boundary integrity is much lower in the CSC/M2 hetero-spheroids. Scale bar = 100 μm. **f** Quantification of the number of migrated cells in the lower chamber of a transwell system indicated that CSC/M2 hetero-spheroids were significantly (*p < 0.05, one-way ANOVA) more migratory compared to CSC mono-spheroids

We then investigated the maintenance of stemness within hetero-spheroids, since we hypothesized that alternative macrophage activation may result in a pro-tumoral reciprocity within hetero-spheroids. Flow analysis for elevated ALDH indicated that M2 hetero-spheroids significantly and robustly (**p < 0.001, one-way ANOVA) increased the maintenance of ALDH+ populations within hetero-spheroids, compared to CSC mono-spheroids (Fig. 3a). Macrophages themselves do not significantly express elevated ALDH in this assay (Additional file 1: Figure S6). A ~ 2 fold increase in ALDH^+^ populations was observed with co-culture with either U937 M2 or PBMC M2 hetero-spheroids, indicating that M2 activation resulted in improved ovarian CSC maintenance. Concomitant with this increase in ALDH, we also observed increased secretion of the IL-6 pro-tumoral cytokine (Fig. 3b). Consequently, inhibition of IL-6 signaling with two small molecule inhibitors, Ruxolitinib or SC144 during the formation of CSC/M2 hetero-spheroids significantly (**p < 0.001, one-way ANOVA) reduced the enrichment of ALDH^+^ cells (Fig. 3c). Our results indicate that the IL-6 signaling axis initiated by M2 macrophage co-culture significantly increases maintenance of ALDH^+^ CSCs within hetero-spheroids.

As a consequence of ALDH^+^ enrichment, CSC/M2 hetero-spheroids were markedly (**p < 0.001, *p* < 0.05, one-way ANOVA) chemoresistant to carboplatin treatment (Fig. 3d). Phase contrast images of drug-treated spheroids demonstrated the loss of integrity of the tight spheroid borders in CSC mono-spheroids, whereas the loss of compactness and integrity was less visually obvious in CSC/M2 hetero-spheroids (Fig. 3d). Concomitant with increased drug resistance, CSC/M2 hetero-spheroids were also significantly (*p < 0.05, one-way ANOVA) more invasive (2–2.6 fold) in transwell assays compared to CSC mono-spheroids (Fig. 3e).

Our experiments indicated that culturing CSCs within CSC/M2 hetero-spheroids resulted in increased maintenance of ALDH^+^ CSC populations, which translated to functional increases in chemoresistance, and invasiveness of CSC/M2 hetero-spheroids. Further, blocking increased IL-6 related signaling in CSC/M2 hetero-spheroids with small molecule inhibitors reduced the maintenance of CSCs within hetero-spheroids.

### Increased WNT signaling in macrophages is responsible for the pro-tumoral and immuno-suppressive phenotype observed in M2-hetero-spheroids

**Fig. 4:**
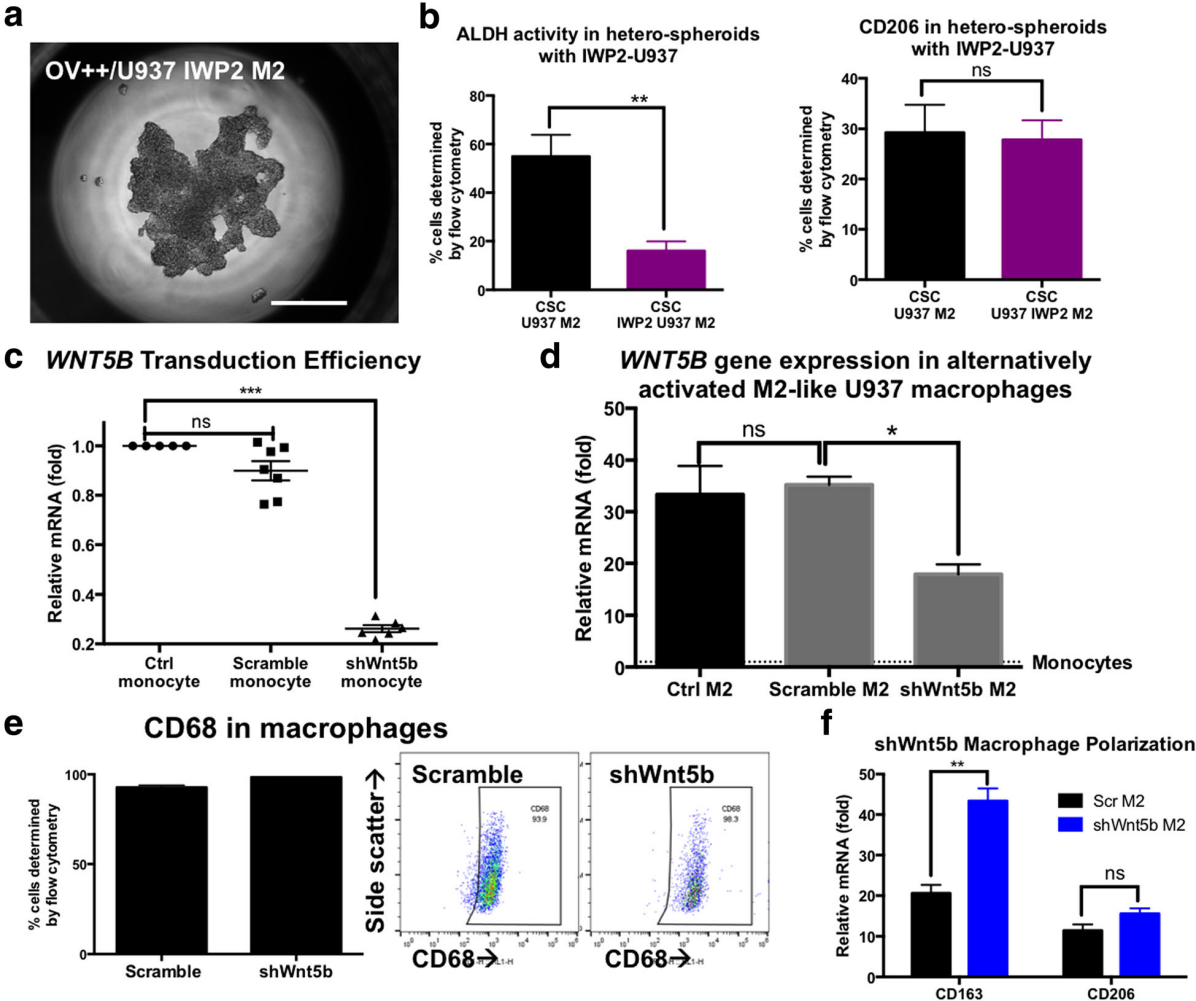
Inhibition of macrophage Wnt-secretion reduces ALDH enrichment in hetero-spheroids. **a** Representative phase contrast image of a hetero-spheroid generated from CSCs and IWP-2 treated U937 M2 macrophages, indicates the formation of aggregated spheroids. Scale bar = 200 μm. **b** Flow analysis revealed that hetero-spheroids with IWP-2 treated M2 macrophages had a significantly diminished ALDH+ enrichment (**p < 0.001, one-way ANOVA) compared to control untreated CSC/M2 hetero-spheroids. However, no change in CD206 expression was observed in hetero-spheroids. **c** Transduction efficiency of monocytes with shRNA directed against *WNT5B* indicated a > 75% efficiency in knockdown of *WNT5B* gene expression in the shWnt5b targeted construct, and minimal changes in *WNT5B* gene expression in the scramble non-targeted lentiviral construct. **d** We utilized shWnt5b monocytes to differentiate and polarize M2 macrophages, and found that there was a 52% reduction in *WNT5B* gene expression in shWnt5b M2 macrophages, compared to scramble or control untreated M2 macrophages, indicating the knockdown of the Wnt5b gene. **e** No changes were observed in CD68 expression in monocytes generated from scramble or sh*WNT5B* treated monocytes, demonstrated by flow analysis and representative plots. **f** Similarly, qPCR analysis of M2 gene expression markers *CD163* and *CD206* indicated increases in both genes, indicative of activation into the M2 program even in shWnt5b macrophages

We hypothesized that similar to CSCs, M2-like macrophage activation may also result in increased WNT signaling. We blocked pan-WNT ligand secretion in M2 macrophages by treatment with IWP-2 following polarization. CSC/IWP2 M2 hetero-spheroids assumed different aggregation morphology and were less compact (Fig. 4a). Furthermore, ALDH expression was significantly lower (**p < 0.001, one-way ANOVA) in CSC/IWP2 M2 hetero-spheroids compared to CSC/M2, with minimal reduction in CD206 expression (Fig. 4b), indicating that macrophage-derived WNT signaling may at least be partially responsible for the phenotypes observed in ovarian CSCs. We investigated the expression of WNT ligands in macrophages, and found *WNT5B* over-expressed ~ 32 fold in M2 macrophages compared to monocytes using qPCR. We used an shRNA plasmid targeting the *WNT5B* ligand (sh-WNT5B) and a non-targeted plasmid (sh-Scramble), and used lentiviral methods to silence *WNT5B* expression in monocytes, with a knockdown efficiency of 76% (Fig. 4c). Macrophages derived from sh-WNT5B were termed sh-WNT5B M2. Despite > 75% knock-down efficiency of *WNT5B* in monocytes, Alternate M2-like activation still resulted in an increase in gene expression of *WNT5B*. However, we found that monocytes treated with sh-WNT5B showed a 52% reduction in WNT5B expression compared to control or scramble M2 macrophages (Fig. 4d). WNT5B knockdown did not alter the CD68^+^ marker expression (Fig. 4e). Treatment with IL-4/M-CSF to induce macrophage polarization resulted in increased gene expression for *CD163* and *CD206*, indicating the development of an alternate M2-like phenotype (Fig. 4f). Therefore, knockdown of *WNT5B* using sh-WNT5B did not significantly alter the differentiation of monocytes to macrophages, or the development of an M2 activated phenotype. However, a significant loss in *WNT5B* gene expression was observed.

### WNT5B is involved in M2 macrophage-CSC interactions, leading to chemoresistance, invasiveness, and increased stemness

**Fig. 5:**
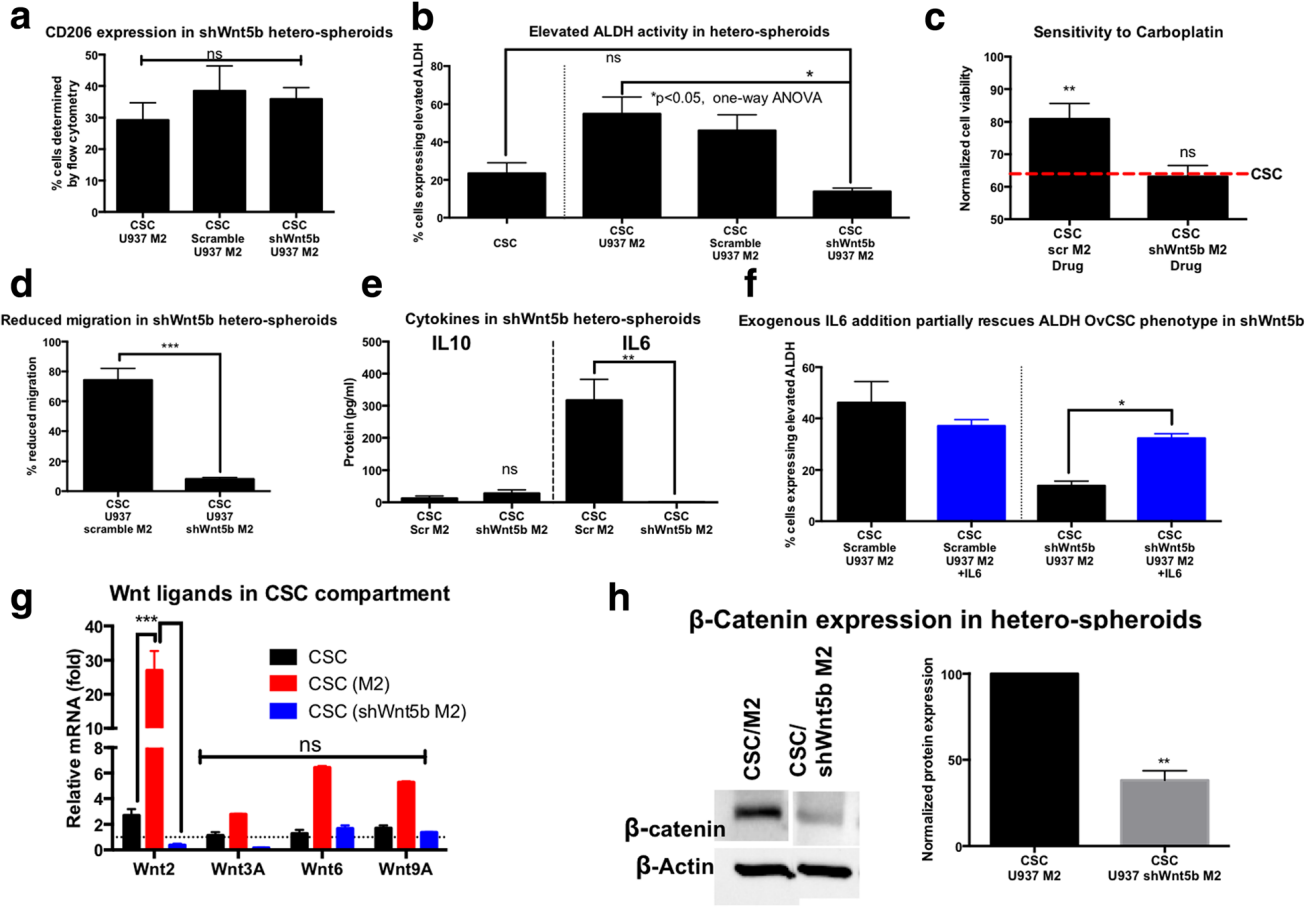
Macrophage knockdown of Wnt5b suppresses ALDH enrichment, with no change in CD206, and increases chemosensitivity of hetero-spheroids*.* **a** Flow analysis of CD206 indicated no significant differences in CSC/M2 and CSC/Scramble M2 and CSC/shWnt5b M2 hetero-spheroids, indicating that CSCs were still capable of maintaining a robust immunosuppressive program in hetero-spheroids, regardless of WNT5B. **b** ALDH elevation however was significantly (*p < 0.05, one-way ANOVA) lowered compared to CSC/U937 M2 hetero-spheroids, and not significantly different (ns) compared to CSC mono-spheroids, indicating that knocking down Wnt5b expression in macrophages reduced ALDH enrichment in hetero-spheroids. **c** Concomitant with the decrease in ALDH, sensitivity to carboplatin also significantly (**p < 0.001, one-way ANOVA) improved, responding to treatment similar to CSC mono-spheroids. The red dashed line indicates the sensitivity levels of CSC mono-spheroids. **d** CSC/shWnt5b M2 hetero-spheroids were also significantly (***p < 0.0001, t-test) less invasive than CSC/Scramble M2 hetero-spheroids. **e** Upon ELISA analysis of secreted IL10 and IL6, we found no changes in IL-10 levels, while we found a stark decrease in secreted pro-tumoral IL-6 cytokine in CSC/shWnt5b M2 hetero-spheroids. **f** Exogenous addition of IL-6 (25 ng/ml) to CSC/shWnt5b M2 hetero-spheroids partially significantly (*p < 0.05, one-way ANOVA) increases ALDH+ enrichment, but does not restore it to levels of original enrichment with CSC/Scramble M2 or CSC/Ctrl M2 hetero-spheroids. **g** In order to identify if there was paracrine macrophage Wnt-driven Wnt signaling in CSCs, we separated the CSCs (using a GFP label) from hetero-spheroids, and probed for gene expression of several Wnt ligands we saw elevated in CSCs compared to bulk OVCAR3. We observed that with M2 macrophage co-culture, several Wnt ligands were upregulated (Wnt2 significantly elevated, ***p < 0.001, two-way ANOVA). However, this elevated gene expression of Wnt ligands was lost in CSCs in hetero-spheroid culture with shWnt5b M2, indicating that Wnt5b was partly responsible for potentiating Wnt signaling within the CSC compartment. **h** This loss in Wnt ligand expression also translated to decreased β-catenin expression in CSC/shWnt5b M2 hetero-spheroids compared to CSC/U937 M2 hetero-spheroids, quantified by densitometry of immunoblots (representative blot shown)

Hetero-spheroids were generated with CSCs and Scramble M2 or sh-WNT5B M2. No significant differences in CD206 expression was observed in these hetero-spheroids, indicating that in co-culture, macrophage knockdown of WNT5B was dispensable for the expression of CD206 (Fig. 5a). However, macrophage knockdown of WNT5B resulted in a significant (*p < 0.05, one-way ANOVA) decrease in ALDH^+^ compartment by over 5-fold, indicating that macrophage-derived WNT5B was crucial for the enrichment of CSC characteristics within hetero-spheroids (Fig. 5b). This reduction in the ALDH^+^ enrichment was also associated with an increased sensitivity to carboplatin (Fig. 5c), and a decreased invasive potential (Fig. 5d). Upon examination of cytokine profiles, we observed no significant differences in IL-10 (Fig. 5e). However, the pro-tumoral CSC-promoting cytokine IL-6 was completely abrogated in the CSC/sh-WNT5B M2 hetero-spheroids, indicating that WNT5B was likely also driving CSC phenotypes through IL-6 secretion. We explored the possibility of restoring the CSC enrichment phenotype by the addition of exogenous IL-6. Upon exogenous addition of IL-6, levels of ALDH^+^ cells increased within CSC/sh-WNT5B M2 hetero-spheroids, indicating that IL-6 is a key effector downregulated upon WNT5B knockdown, but may likely not be the only mediating factor in promoting CSC maintenance via WNT5B (Fig. 5f). Concomitant with the loss of IL-6 in CSC/sh-WNT5B M2 hetero-spheroids, we also observed reduced phosphorylated STAT3 (42.4 ± 5.5%) in immunoblots compared to CSC/M2 hetero-Additional file 1: Figure S7). Lastly, we explored the possibility of WNT-driven WNT signaling in CSCs, in response to macrophage WNT5B. We observed through gene expression analysis that several WNT ligands were significantly over-expressed in CSCs co-cultured with M2 macrophages, and there was a loss in WNT ligand expression upon co-culture with sh-WNT5B M2 macrophages (Fig. 5g). Consequently, there was also an associated ~ 50% loss of β-catenin protein expression in CSC/sh-WNT5B M2 hetero-spheroids, indicating a lower paracrine WNT activation in CSCs in co-culture with macrophages where WNT5B was knocked down (Fig. 5h).

Overall, we observed that although knockdown of macrophage *WNT5B* did not alter macrophage M2 phenotype, macrophage-derived WNT5B proved to be a driver of WNT-activated WNT signaling and IL-6 secretion, responsible partly for the increased CSC phenotypes observed in CSC/M2 hetero-spheroids.

### CSCs conditioned in M2 hetero-spheroid culture are more tumorigenic in vivo, and shWNT5B macrophage conditioning inhibits tumorigenicity

**Fig. 6:**
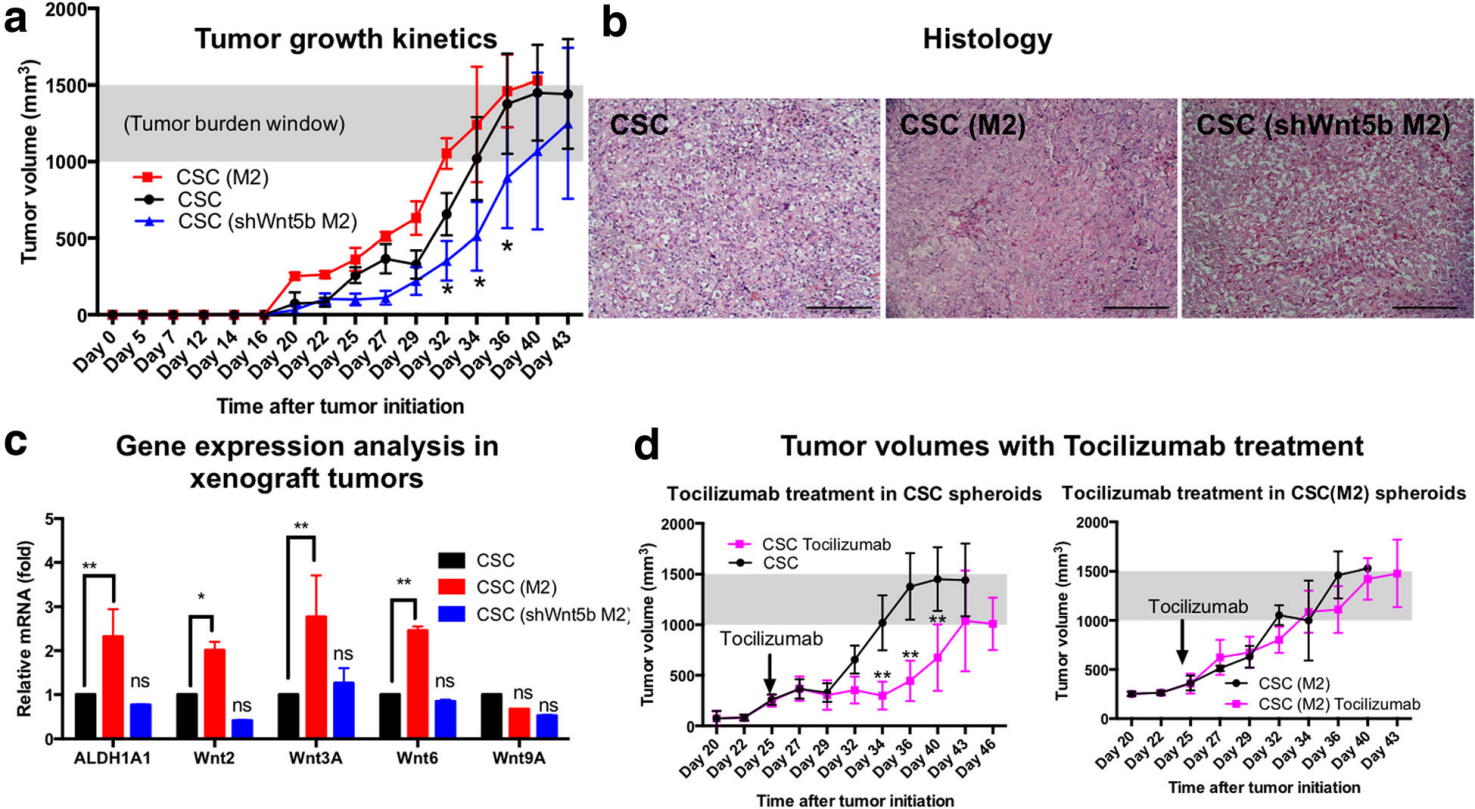
Changes in tumorigenicity of hetero-spheroids in vivo in NSG mice. Tumors were generated in NSG mice from CSC mono-spheroids, or CSCs isolated from hetero-spheroids in CSC/M2 or CSC/shWNT5b M2. **a** Tumor initiation and growth kinetics are shown for all three groups, where CSC(M2) tumors (red curve) demonstrates elevated tumorigenicity, with faster tumor initiation and tumor burden development (gray shaded area indicates the tumor burden window). Similarly, the blue trace indicates the tumor growth in CSC/shWNT5b M2 tumors, which is significantly (*p < 0.05, two-way ANOVA) lower and slower compared to CSC and CSC(M2) groups at the time points indicated. **b** Histological examination demonstrates that all groups establish similar structures of tumors sub-cutaneously, with CSC tumors and CSC (M2) tumors establishing dense epitheliod cells, with CSC (shWNT5b M2) tumors more loosely arranged. Scale bar = 100 μm. **c** Gene expression analysis of xenografted tumors indicated that an elevated *ALDH1A1* expression was maintained in CSC(M2) tumors (**p < 0.001, two-way ANOVA). M2-like macrophage conditioning also helped CSCs retain elevated expression of *WNT2, WNT3A and WNT6* (*p < 0.05, **p < 0.001, two-way ANOVA) compared to CSCs in mono-spheroids. All other genes and conditions were not significantly different (ns) compared to CSCs, specifically CSC/shWnt5B M2 tumors. **d** The human IL-6 inhibitor Tocilizumab was used intra-peritoneally on CSC and CSC(M2) tumors, once palpable tumors were observed (Day 25), indicated by the arrow. Tocilizumab treatment resulted in a significant decrease in tumor volume (**p < 0.001, two-way ANOVA) compared to control untreated tumors in CSC mono-spheroid tumors (compare purple trace to black trace; ~ 70% reduction in tumor burden). However, in CSC/M2 tumors, tocilizumab treatment did not significantly alter the growth kinetics of treated vs. untreated tumors (~ 24% reduction in tumor burden), indicating the detainment of a chemoresistant phenotype upon M2-like macrophage co-culture and conditioning. Furthermore, the treated CSC Tocilizumab condition also resembles the control CSC (shWNT5B M2) tumors (blue trace in A), indicating that partial loss or inhibition of IL-6 based CSC enrichment may be at play

Three tested conditions: CSC (mono-spheroids), CSC (M2; conditioned in hetero-spheroids) and CSC(sh-WNT5B M2; conditioned in hetero-spheroids) generated tumors in NSG mice (Fig. 6a). The rate of tumor initiation and kinetics of growth was significantly different between all three conditions, with CSC(M2) tumors initiating at the fastest rate of 11.97mm^3^/day. CSC(shWNT5b M2) tumors were significantly slower to initiate at 4.74mm^3^/day, compared to CSC tumors at 5.89mm^3^/day. CSC(M2) tumors reached the maximum tumor burden window earlier, compared to CSC and CSC(shWNT5B M2) tumors (Fig. 6a). Histologic examination of the xenografts reveals that the lesions are composed of solid sheets of tumor cells with epithelioid morphology. In the CSC group, dense sheets of polygonal tumor cells with abundant cytoplasm and conspicuous nuclei are noted, with little intercellular space (Fig. 6b). In the CSC(M2) group, sheets of large epithelioid tumor cells with abundant eosinophilic cytoplasm constitute the entire tumor with little intercellular space and stroma. In the CSC(shWNT5B M2) group, the tumor cells are much smaller and loosely arranged in cords and strands. Occasional apoptotic cells are seen with a low mitotic count. Gene expression levels were assessed in xenografted tumors from the three groups, to understand if macrophage conditioning was maintained in vivo (Fig. 6c). Our data indicated that an elevated *ALDH1A1* expression was retained significantly in CSC(M2) tumors (**, *p* < 0.001, two-way ANOVA), compared to CSC(shWNT5B M2) tumors (ns; not significant). Similarly, elevated Wnt ligands observed in vitro upon CSC/macrophage co-culture in hetero-spheroids (Fig. 5g) was re-evaluated in xenografts. We observed the maintenance of a significant elevation in Wnt2, Wnt3A, and Wnt6 (*p < 0.05, **p < 0.001, two-way ANOVA) in CSC(M2) tumors compared to CSC tumors. CSC(shWNT5B M2) tumors were not significantly different in Wnt ligand elevation compared to CSC tumors. Furthermore, we tested the effect of the human IL-6 inhibitor Tocilizumab on CSC and CSC(M2) spheroids (Fig. 6d). CSC spheroids were significantly responsive to Tocilizumab treatment, evident from the divergent treatment curve from the control untreated group (purple curves, Fig. 6d). CSC(M2) tumors were however significantly resistant to Tocilizumab treatment, indicating the elevated IL6 conditioning activity, in line with elevated *ALDH1A1* gene expression and elevated Wnt ligand expression. Change in tumor burden with Tocilizumab treatment in CSC spheroids ranged between 67.7–70.7%, while in CSC(M2) tumors, the reduction was merely 23.8–24% of control tumor volumes.

## Discussion

Macrophages play an important role in tissue homeostasis, with tumor-associated macrophages promoting cancer progression, metastasis, angiogenesis and tumorigenicity. Tumor-associated macrophages are a major fraction of cells within the epithelial ovarian cancer ascites microenvironment [23, 24], and are responsible for recurrence and metastasis of ovarian cancer, including promotion of resistance and the preservation of a more de-differentiated, i.e. stem-like cancer cell phenotype [7, 25]. Given the enriched presence of CSCs and the abundant presence of macrophages within the malignant ascites [1, 2, 11], we opted to use a hetero-spheroid model to bring both these cells in close association within one 3D structure in vitro. The primary advantage of this model is that it facilitates cell-cell interactions in a 3D microenvironment [13–15]. Therefore, the hanging drop spheroid model is uniquely positioned to understand how ovarian CSCs and macrophages interact with each other (simulating their presence within the ascites), and specifically, test our hypotheses: i) CSCs activate macrophages differently comparing to the bulk ovarian cancer cells; and ii) the activated macrophages promote functional CSC phenotypes.

In our experiments, we observed similar levels of differentiation and subsequent activation of macrophages by using the 3D platform, compared to monocytes differentiated and activated traditionally in 2D tissue culture plates by us (Additional file 1: Figure S1) and others [26–28]. Interestingly, along with clear hallmarks of alternate macrophage activation (IL-10 secretion, *CD163* and *CD206* gene expression), we also observe an elevated macrophage secretion of IL-6, which is not typically associated with M2 macrophages, but is observed in bipolar tumor associated macrophage populations in the malignant ascites of ovarian cancer [11, 29].

In CSC/macrophage hetero-spheroids, we find that CD68^+^ macrophages make up ~ 20% of the population at day 5, even though they start at 50% initially, likely due to CSC proliferation taking over the spheroid. Our results suggest that CSCs have an intrinsically higher immuno-suppressive program, driving CD206 expression in M0 macrophages to a larger extent than bulk unsorted ovarian cancer cells, evident also by the increased *IL10* gene expression in CSCs. This observation is in line with results observed in literature where ovarian cancer cells activate macrophages to alternative M2-like pro-tumoral phenotype [9, 30]. Indeed, macrophages exposed to IL-10 are known to polarize into M2 phenotypes [31, 32] under sustained IL-10 exposure, and CSCs from ovarian and other cancers can induce pro-tumoral macrophage phenotypes through other pathways including NF-kB [33, 34].

Consequently, we determined that hetero-spheroids with alternatively activated M2-like macrophages increased maintenance of the ALDH^+^ CSC populations. This is in line with observations in ovarian, breast and hepatocellular carcinomas, where tumor-associated macrophages induce stemness and increase CSCs [25, 35, 36]. In our experiments, ALDH^+^ increase could also be correlated with increased levels of the cytokine IL-6 in M2 hetero-spheroids. The involvement of M2-derived IL-6 is further strengthened by the fall in ALDH^+^ enrichment with inhibitors of the IL-6/STAT3 signaling axis (Ruxolitinib, SC144, and Tocilizumab). The increase in ALDH^+^ enrichment and the emergence of CD206^+^ macrophages was also observed in CSC/M0-M2 hetero-spheroids generated from an additional high-grade serous cell line, Kuramochi (Additional file 1: Figure S8) and tumor cells and CSCs from a high-grade serous primary ovarian carcinoma sample Additional file 1: Figure S9).

The WNT/β-catenin pathway is heavily implicated in the maintenance of CSCs in ovarian cancer, so much so, that it is an attractive therapeutic target [37–39]. Hence, the elevation of several *WNT* ligands in CSCs compared to bulk OVCAR3 cancer cells is unsurprising, as we observe in our results. It is however likely, that some of the CSC-derived WNT ligands could potentially drive M2-like alternate macrophage activation, as evidenced in other pathologies where the involvement of WNT3A, WNT6 and other WNT ligands drive alternative macrophage activation [20, 40].

In our experiments, we found that macrophage-specific inhibition of WNT secretion resulted in a significant reduction in ALDH^+^ enrichment in hetero-spheroids, implying that WNT signaling was likely involved bi-directionally in CSC/macrophage interactions. The lone WNT ligand we found significantly upregulated in M2-like macrophages (compared to monocytes or M0 macrophages) was *WNT5B*, in line with transcriptomic and gene expression analyses performed on human M2 macrophages by other groups [41, 42]. Interestingly, macrophage *WNT5B* knockdown resulted in the loss of ALDH^+^ enrichment in the CSC compartment of hetero-spheroids, with an associated loss of IL-6 secretion. In other pathogenic states, activation of WNT5B is associated with IL-6 secretion [43, 44], and hence, it is not surprising that we observe the loss in IL-6 upon *WNT5B* knockdown. Exogenous addition of IL-6 partially restores elevated ALDH levels in CSC/sh-WNT5B M2 hetero-spheroids, indicating that IL-6 is at least partly responsible for the observed phenotype. Additionally, we also observed that several *WNT* ligands were upregulated in the CSC compartment of hetero-spheroids cultured in CSC/M2 compared to CSC mono-spheroids, and the upregulation dropped in CSCs cultured in CSC/sh-Wnt5b M2 hetero-spheroids. Tumor stroma-derived WNT ligands like WNT3 and WNT5B are critical factors that instigate invasive behavior, and induction of an EMT phenotype in tumor epithelial cells [45]. In fact, WNT5B associated exosomes promoted cancer cell migration and proliferation in a paracrine manner [46].

This points to enhanced Wnt signaling within the CSC compartment upon co-culture with alternatively activated M2-like macrophages, likely mediated by secreted Wnt (specifically also WNT5B) arising from the macrophage compartment, in line with several observations where tumor associated/M2 macrophages increase Wnt signaling in epithelial cells [22, 47–49].

The tumor-intrinsic WNT-β-catenin signaling is shown to promote a T-cell exclusion phenotype [18]. Utilizing a robust immune deconvolution algorithm, we analyzed high-grade serous carcinomas and found a consistent phenotype, suggesting that the tumor-intrinsic WNT-β-catenin activation may promote immune suppression across cancer types (Additional file 1: Figure S10). However, the cellular mechanism underpinning this link remained less characterized. This study employs a high-fidelity 3D culture model to reveal the reverse link between the expression levels of *WNT5B* in alternatively activated macrophages and cancer cells, possibly leading to T-cell exclusion. However, it is important to note that the WNT signaling pathway has pleotropic effects on a broad range of cell types including several types of immune cells [50, 51]. Recent studies show that Wnt pathway is also crucial for the development of T-cells [52]. The Wnt-β-catenin pathway maintains the multipotency of memory CD8^+^ T-cells with anti-tumor properties [53]. Thus, dosing, treatment schedule, and relatively more specific tumor-targeted delivery approaches are likely important to unleash the potential of the immune-priming properties of WNT-β-catenin inhibitors.

**Fig. 7:**
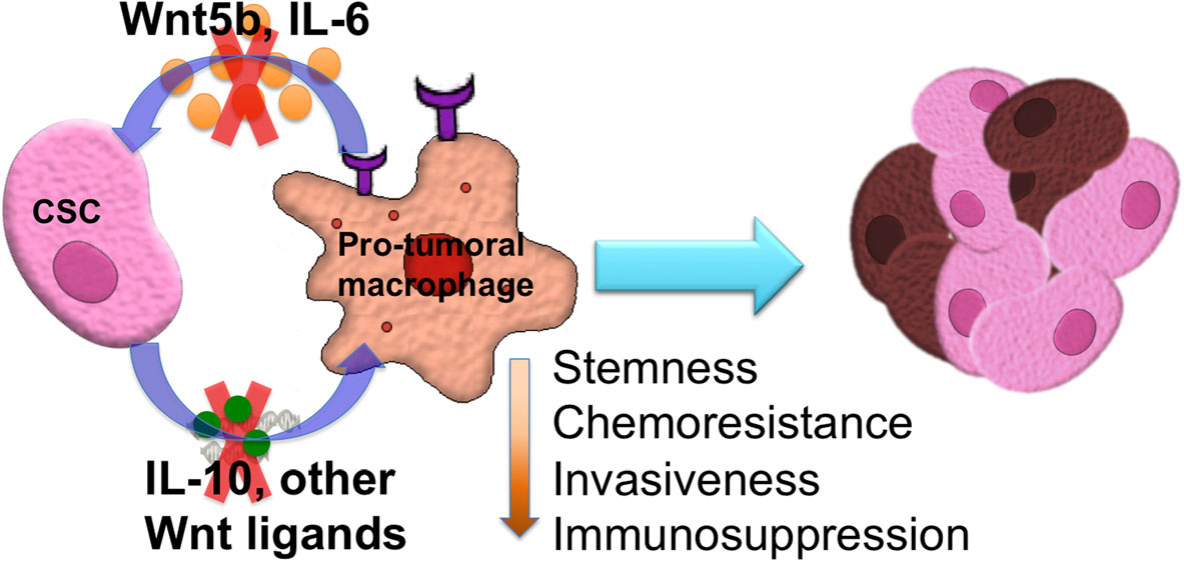
Ovarian cancer stem cells and macrophages reciprocally interact through the Wnt pathway to promote a pro-tumoral microenvironment. Our data suggests that in hetero-spheroids, CSCs drive a CD206^+^ expressing macrophage phenotype, suggestive of pro-tumoral M2 activation through secretion of the immuno-suppressive cytokine IL-10, and through WNT ligands. We also observe that macrophage-derived WNT ligands, specifically WNT5B, and to some extent the pro-tumoral cytokine IL-6, drive an enrichment in ALDH^+^ cells within hetero-spheroids. Knockdown of macrophage WNT5B expression, or inhibiting downstream activation of IL-6 using Ruxolitinib or sc144, result in a significant loss of ALDH^+^ populations within hetero-spheroids. Importantly, we find that the Wnt pathway is involved bi-directionally in CSC-macrophage interactions, and can potentially be targeted to reduce stemness, chemoresistance, invasiveness and immunosuppression in ovarian cancer

In conclusion, we demonstrate a robust hetero-spheroid culture system, where ovarian CSCs and activated macrophages can be brought in close association in a 3D in vitro microenvironment, simulating their non-adherent presence within malignant ascites. We demonstrate the presence of macrophages within spheroids and their pro-tumoral activation by CSCs. Reciprocally, pro-tumoral activated macrophages also promote a chemoresistant and invasive phenotype of the CSC compartment and its enrichment within hetero-spheroids, potentially contributing to highly malignant and metastatic disease (Fig. 7). Lastly, we were able to understand the reciprocal involvement of the WNT pathway, with activation of paracrine WNT signaling by macrophages in ovarian CSCs, with wide-implications for new therapeutic targets to specifically eradicate the immuno-modulation of macrophages by CSCs that contribute to recurrent disease.

## Additional file

Additional file 1: **Figure S1.** U937 monocytes differentiated in 3D hanging drop arrays are equivalent to U937 monocytes differentiated in 2D. **Figure S2.** No change in proliferation in CSC compartments of hetero-spheroids. **Figure S3.** Gating strategy for Flow cytometry. **Figure S4.** Cancer cells do not significantly express the macrophage marker, CD206. **Figure S5** CD163 expression is elevated in CSC/U937 M2 hetero-spheroids. **Figure S6.** Macrophages do not significantly express elevated ALDH. **Figure S7.** phospho-STAT3 is significantly reduced in CSC/shWNT5B-M2 hetero-spheroids compared to CSC/M2 hetero-spheroids. **Figure S8.** Kuramochi-CSC also drive elevated CD206 expression in macrophages, and polarized macrophages enrich ALDH+ cells in Kuramochi CSC and resistance to carboplatin. **Figure S9.** High-grade serous ovarian cancer Patient 259 derived CSC drive elevated CD206 expression in macrophages, and demonstrate a carboplatin resistant phenotype. **Figure S10.** Scatter plots for correlation of WNT5B with immune cell subsets in ovarian carcinoma. **Table S1.** List of primers used for qPCR experiments. (ZIP 1916 kb)

## Data Availability

Please contact corresponding author for data requests.
