# Supplementary material for: Ovarian cancer stem cells and macrophages reciprocally interact through the WNT pathway to promote pro-tumoral and malignant phenotypes in 3D engineered microenvironments
Source: J Immunother Cancer. 2019 Jul 19;7:190. doi: 10.1186/s40425-019-0666-1 (PMC6642605; doi:10.1186/s40425-019-0666-1)
Supplement: Supplementary file 1 — Figure S1. U937 monocytes differentiated in 3D hanging drop arrays are equivalent to U937 monocytes differentiated in 2D. Figure S2. No change in proliferation in CSC compartments of hetero-spheroids. Figure S3. Gating strategy for Flow cytometry. Figure S4. Cancer cells do not significantly express the macrophage marker, CD206. Figure S5 CD163 expression is elevated in CSC/U937 M2 hetero-spheroids. Figure S6. Macrophages do not significantly express elevated ALDH. Figure S7. phospho-STAT3 is significantly reduced in CSC/shWNT5B-M2 hetero-spheroids compared to CSC/M2 hetero-spheroids. Figure S8. Kuramochi-CSC also drive elevated CD206 expression in macrophages, and polarized macrophages enrich ALDH+ cells in Kuramochi CSC and resistance to carboplatin. Figure S9. High-grade serous ovarian cancer Patient 259 derived CSC drive elevated CD206 expression in macrophages, and demonstrate a carboplatin resistant phenotype. Figure S10. Scatter plots for correlation of WNT5B with immune cell subsets in ovarian carcinoma. Table S1. List of primers used for qPCR experiments. (ZIP 1916 kb) [file 40425_2019_666_MOESM1_ESM.zip › Supplementary Information - Rev1.docx]

**Ovarian cancer stem cells and macrophages reciprocally interact through the WNT pathway to promote pro-tumoral and malignant phenotypes in 3D engineered microenvironments**

Shreya Raghavan, Pooja Mehta, Yuying Xie, Yu L. Lei, Geeta Mehta

**Supplementary Information:**

**Supplementary Figure 1: U937 monocytes differentiated in 3D hanging drop arrays are equivalent to U937 monocytes differentiated in 2D**

U937 monocytes were plates onto regular tissue culture treated dishes, and treated with 5ng/ml PMA, and activated with 20ng/ml IL4 and 20ng/ml MCSF. Similarly, monocytes were plated onto hanging drop arrays and treated with the same amounts of PMA, IL4 and MCSF.

(A): Representative phase contrast images show differentiated and activated macrophages in 2D and 3D, scale bar = 200 µm. At the end of 72 hours, cells were harvested and RNA was isolated using protocols mentioned in the main manuscript.

(B): Equivalent levels of *CD163*, and *CD206* gene expression were observed. Importantly, similar levels of *WNT5B* gene expression was observed between conventional 2D activation, and 3D activation, indicating that the hanging drop array is a good non-adherent methodology to generate differentiated and activated macrophages.

(C) An arginase activity assay (Abcam) was performed on M0, 2D activated M2 and 3D activated M2 U937 macrophages, following manufacturer’s protocols. 3D activated M2 macrophages demonstrated a significantly (**, p<0.01, one-way ANOVA) higher arginase activity compared to M0 differentiated macrophages, indicating the functionality of IL4 and MCSF polarized M2 macrophages.

**Supplementary Figure 2: No change in proliferation in CSC compartments of hetero-spheroids**

In order to measure proliferation, we used GFP-tagged OVCAR3 CSCs to generate mono- or hetero-spheroids. At the end of Day 5, spheroids were harvested and fixed in ice cold methanol for 1 hour, and stained with the Ki67 antigen. Flow cytometry was used to analyze GFP^+^ Ki67^+^ populations indicative of actively proliferating CSCs. Our data indicated no significant differences in proliferation of CSCs in hetero-spheroids with U937 macrophages, compared to CSC mono-spheroids.

**Supplementary Figure 3: Gating strategy for Flow cytometry**

Representative flow cytometry plots including an isotype control plot for each fluorophore used is demonstrated. Hand-drawn gates were used to follow the shape of the density plots observed, and were identical between the isotype control, and test sample for each fluorophore. Gates were derived using the isotype control antibodies or DEAB with background fluorescence restricted to under 0.5% - the same gate was subsequently utilized for the test sample. (A): Representative gate for the APC-isotype control antibody, and the identical gate on a CD68-APC antibody; (B): Representative gate for the PE-isotype antibody and the CD206-PE antibody on a test sample; (C): Two representative gates to demonstrate the gating strategy to identify elevated ALDH activity, using DEAB inhibited ALDH fluorescence.

**Supplementary Figure 4: Cancer cells do not significantly express the macrophage marker, CD206**

We used flow cytometry analysis to measure the amounts of the alternatively activated macrophage marker, CD206 in bulk cancer cells or CSCs. We found <1% of cells expressing a background level of CD206 in both the OVCAR3 and Kuramochi, high grade serous cell lines.

**Supplementary Figure 5: CD163 expression is elevated in CSC/U937 M2 hetero-spheroids**

We performed flow cytometry analysis for the alternatively activated macrophage marker CD163. (A) Representative flow analysis plots indicating an elevated expression of CD163 in CSC/U937 M2 hetero-spheroids, compared to CSC only mono-spheroids. (B) Quantification of CD163 indicated a significant (**p<0.001, t-test) elevation in hetero-spheroids.

**Supplementary Figure 6:** **Macrophages do not significantly express elevated ALDH**

U937 macrophages (M0 and M2) were harvested and subject to flow analysis for elevated ALDH – neither expressed any significant levels of elevated ALDH (<1%).

**Supplementary Figure 7: phospho-STAT3 is significantly reduced in CSC/shWNT5B-M2 hetero-spheroids compared to CSC/M2 hetero-spheroids**

Protein lysates from CSC/shWNT5B M2 and CSC/M2 hetero-spheroids were resolved on a 4-20% polyacrylamide gel, following protocols described in Section 2.10. Resolved and transferred proteins were blocked with 5% Bovine Serum Albumin, and incubated in primary antibodies directed against phospho-STAT3 Tyr 705 (Cell Signaling Technologies), total STAT3 (Cell Signaling Technologies), and/or β-Actin (Sigma Aldrich) diluted in 3% bovine serum albumin, and with appropriate HRP-conjugated secondary antibodies. Blots were imaged and band densities were quantified using Image J. Following normalization to β-Actin as a loading control, we determined that CSC/shWNT5B M2 hetero-spheroids had a 42% reduction in phospho-STAT3 compared to CSC/M2 hetero-spheroids, indicating lowered activation of pSTAT3 triggered by shWNT5B M2 co-culture.

**Supplementary Figure 8:** **Kuramochi-CSC also drive elevated CD206 expression in macrophages, and polarized macrophages enrich ALDH+ cells in Kuramochi CSC and resistance to carboplatin**

Hetero-spheroids were generated from another high grade serous ovarian cancer cell line (Kuramochi/Kura) and CSCs from the Kuramochi cell line (Kuramochi CSC or Kura+). (A): Representative flow cytometry plots indicating that Kuramochi CSCs also drive and maintain CD206 expression in M0 macrophages to higher extents than unsorted bulk Kuramochi cells; (B) Quantification of flow cytometry analysis indicated a significant elevation in CD206 expression in Kura CSC/M0 hetero-spheroids compared to Kura/M0 heter-spheroids (*p<0.05, two-way ANOVA). (C) Representative flow cytometry plots indicating an increase in ALDH^+^ populations in Kuramochi CSC/M2 hetero-spheroids compared to Kuramochi CSC mono-spheroids; (D) Representative phase contrast images of carboplatin treated Kuramochi CSC and Kuramochi CSC/M2 hetero-spheroids; Phase contract images of carboplatin treated Kuramochi CSC spheroids show the complete loss of boundary integrity in mono-spheroids, compared to the maintenance of a compact spheroid structure indicative of resistance in Kuramochi CSC/M2 hetero-spheroids; Scale bar = 200µm. (E) Quantification of viability indicated a significant (**p<0.001, one-way ANOVA) increase in resistance to carboplatin.

**Supplementary Figure 9: High-grade serous ovarian cancer Patient 259 derived CSC drive elevated CD206 expression in macrophages, and demonstrate a carboplatin resistant phenotype.**

Patient 259 (high grade serous ovarian carcinoma) derived tumor cells, and CSC were utilized to generate hetero-spheroids: (A) Representative flow cytometry plots comparing alternatively activated macrophage marker CD206 in Pt259 Bulk/U937 M0 and Pt259 CSC/U937 M0 spheroids; Quantification indicated that CD206 expression was significantly (**p<0.01, one-way ANOVA) higher in Pt259 CSC/M0 hetero-spheroids, implying that CSCs drive a higher immune-suppressive program in M0 macrophages compared to bulk tumor cells.

(B) Furthermore, co-culture with IL4-activated M2 macrophages significantly elevated ALDH^+^ populations (*p<0.05, one-way ANOVA) in hetero-spheroids, and CSC enrichment was absent in hetero-spheroids generated from U937 shWnt5b M2 macrophages, where Wnt5b gene expression was knocked down.

(C) Additionally, Pt259 CSC/M2 hetero-spheroids were significantly more resistant to carboplatin compared to mono-spheroids. Phase contrast images indicate the loss of boundary integrity in spheroids responsible to carboplatin (Pt259 CSC and Pt259 CSC/shWNT5b M2 spheroids). Scale bar= 200µm. Quantification of viability indicated significantly elevated resistance in Pt259 CSC/M2 hetero-spheroids (***p<0.001, one-way ANOVA) compared to Pt259 mono-spheroids, and a reduction in carboplatin resistance in Pt259 CSC/shWNT5B M2 hetero-spheroids compared to Pt259/M2 hetero-spheroids.

**Supplementary Figure 10: Scatter plots for correlation of WNT5B with immune cell subsets in ovarian carcinoma**

We downloaded the microarray datasets for TCGA high-grade serous carcinoma from Broad GDAC Firehose (<https://gdac.broadinstitute.org/>). We then use FARDEEP, a deconvolution method based on adaptive least trimmed square, to estimate the abundance of the M1-like macrophage, γδ T-cells, activated NK cells and activated CD4^+^ memory T-cells in ovarian carcinoma. The correlations and corresponding p-values between the expression levels of *WNT5B* and the indicated immune cell subsets were calculated in R, and the scatter plots were generated in R using the ggplot2 package.

Scatter plots are shown to demonstrate the correlation coefficient of *WNT5B* with various immune subsets in ovarian cancer. *WNT5B* was strongly negatively correlated with M1-like macrophages (-0.154; p 0.008) indicating a negative association with anti-tumor inflammatory activity. In agreement, *WNT5B* was also negatively correlated with CD4^+^ memory cells (-0.139, p 0.016), activated NK cells (-0.121, p 0.037), and γδ-T cells (-0.127, p 0.028).

**Supplementary Table 1: List of primers used for qPCR experiments**

Primers for the following genes are listed: *GAPDH, CD163, CD206, IL10, WNT5B, WNT2, WNT3, WNT3A, WNT6, WNT9A, ALDH1A1*.
