# Supplementary figures and images for: Ovarian cancer stem cells and macrophages reciprocally interact through the WNT pathway to promote pro-tumoral and malignant phenotypes in 3D engineered microenvironments
Source: J Immunother Cancer. 2019 Jul 19;7:190. doi: 10.1186/s40425-019-0666-1 (PMC6642605; doi:10.1186/s40425-019-0666-1)

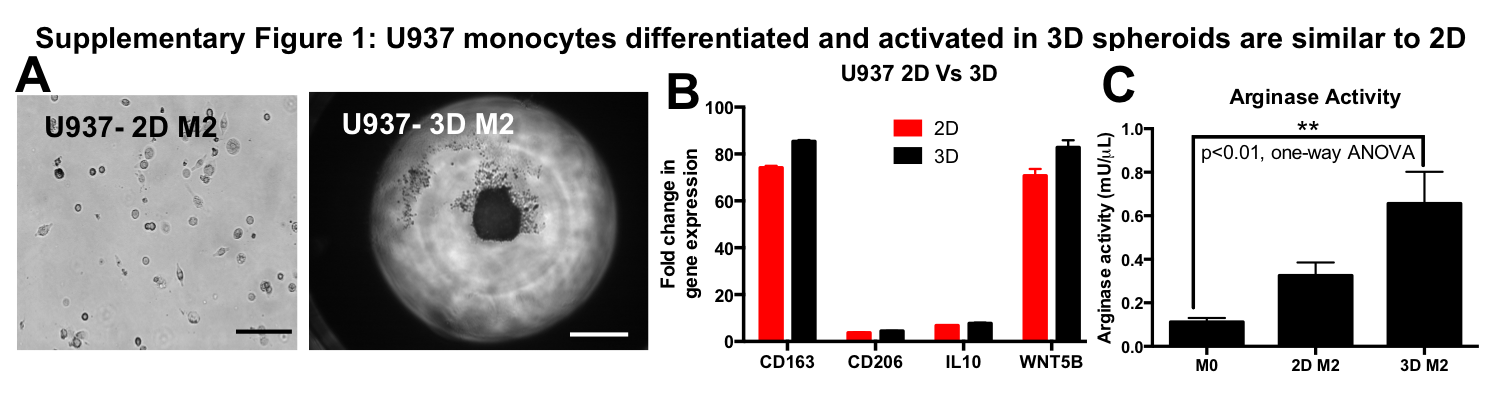

Supplement: Supplementary file 1 — Figure S1. U937 monocytes differentiated in 3D hanging drop arrays are equivalent to U937 monocytes differentiated in 2D. Figure S2. No change in proliferation in CSC compartments of hetero-spheroids. Figure S3. Gating strategy for Flow cytometry. Figure S4. Cancer cells do not significantly express the macrophage marker, CD206. Figure S5 CD163 expression is elevated in CSC/U937 M2 hetero-spheroids. Figure S6. Macrophages do not significantly express elevated ALDH. Figure S7. phospho-STAT3 is significantly reduced in CSC/shWNT5B-M2 hetero-spheroids compared to CSC/M2 hetero-spheroids. Figure S8. Kuramochi-CSC also drive elevated CD206 expression in macrophages, and polarized macrophages enrich ALDH+ cells in Kuramochi CSC and resistance to carboplatin. Figure S9. High-grade serous ovarian cancer Patient 259 derived CSC drive elevated CD206 expression in macrophages, and demonstrate a carboplatin resistant phenotype. Figure S10. Scatter plots for correlation of WNT5B with immune cell subsets in ovarian carcinoma. Table S1. List of primers used for qPCR experiments. (ZIP 1916 kb) [file 40425_2019_666_MOESM1_ESM.zip › SupFig1.tif]

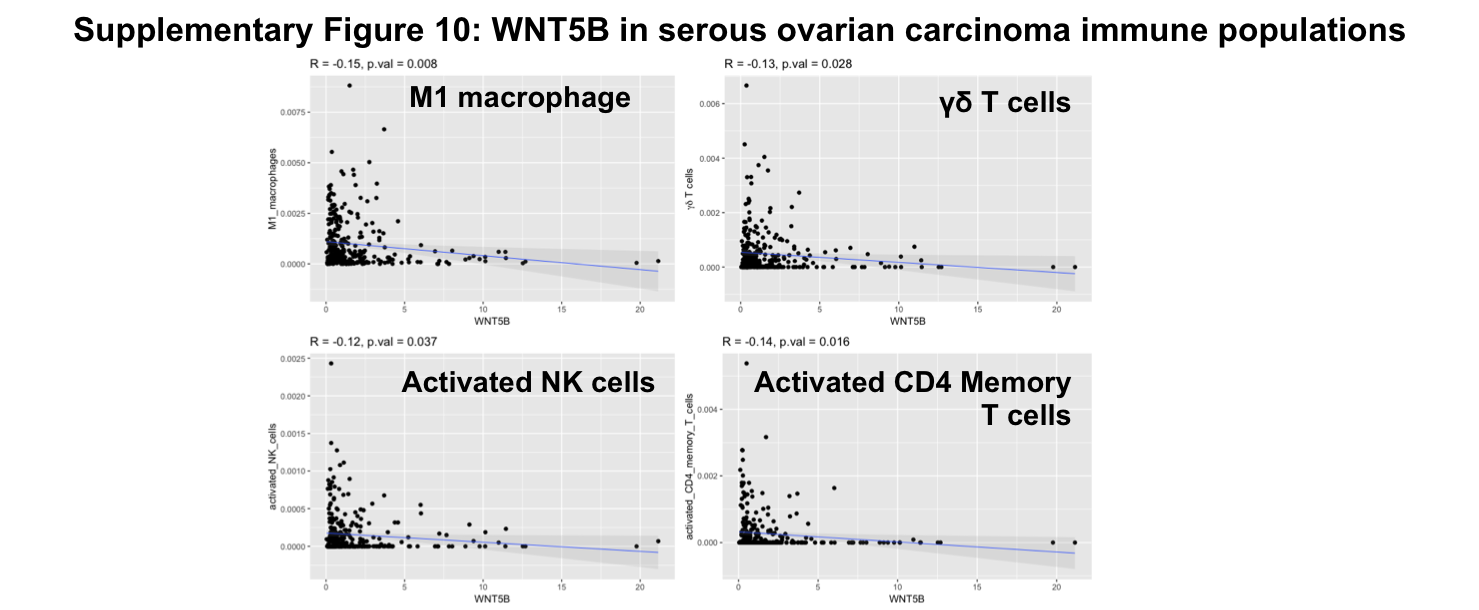

Supplement: Supplementary file 1 — Figure S1. U937 monocytes differentiated in 3D hanging drop arrays are equivalent to U937 monocytes differentiated in 2D. Figure S2. No change in proliferation in CSC compartments of hetero-spheroids. Figure S3. Gating strategy for Flow cytometry. Figure S4. Cancer cells do not significantly express the macrophage marker, CD206. Figure S5 CD163 expression is elevated in CSC/U937 M2 hetero-spheroids. Figure S6. Macrophages do not significantly express elevated ALDH. Figure S7. phospho-STAT3 is significantly reduced in CSC/shWNT5B-M2 hetero-spheroids compared to CSC/M2 hetero-spheroids. Figure S8. Kuramochi-CSC also drive elevated CD206 expression in macrophages, and polarized macrophages enrich ALDH+ cells in Kuramochi CSC and resistance to carboplatin. Figure S9. High-grade serous ovarian cancer Patient 259 derived CSC drive elevated CD206 expression in macrophages, and demonstrate a carboplatin resistant phenotype. Figure S10. Scatter plots for correlation of WNT5B with immune cell subsets in ovarian carcinoma. Table S1. List of primers used for qPCR experiments. (ZIP 1916 kb) [file 40425_2019_666_MOESM1_ESM.zip › SupFig10.tif]

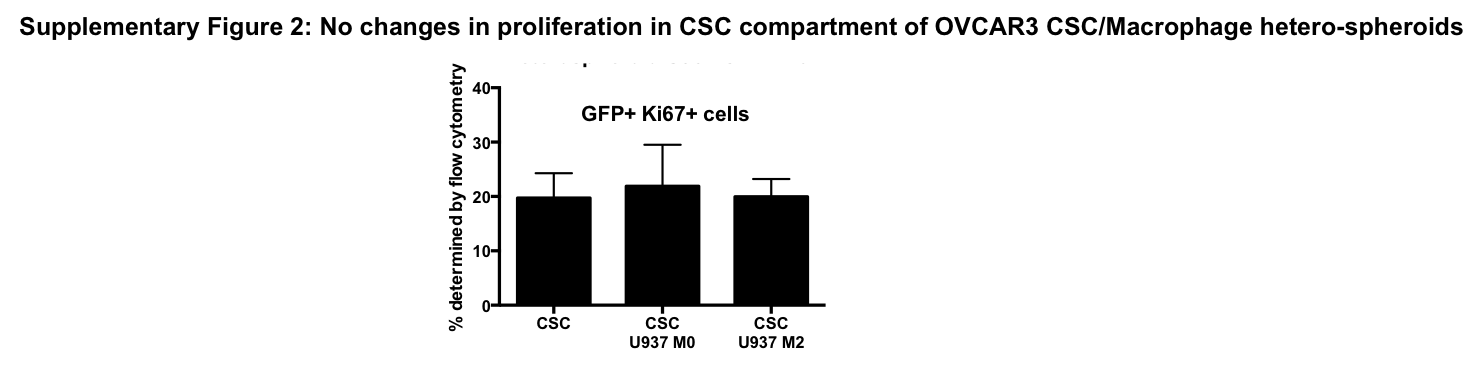

Supplement: Supplementary file 1 — Figure S1. U937 monocytes differentiated in 3D hanging drop arrays are equivalent to U937 monocytes differentiated in 2D. Figure S2. No change in proliferation in CSC compartments of hetero-spheroids. Figure S3. Gating strategy for Flow cytometry. Figure S4. Cancer cells do not significantly express the macrophage marker, CD206. Figure S5 CD163 expression is elevated in CSC/U937 M2 hetero-spheroids. Figure S6. Macrophages do not significantly express elevated ALDH. Figure S7. phospho-STAT3 is significantly reduced in CSC/shWNT5B-M2 hetero-spheroids compared to CSC/M2 hetero-spheroids. Figure S8. Kuramochi-CSC also drive elevated CD206 expression in macrophages, and polarized macrophages enrich ALDH+ cells in Kuramochi CSC and resistance to carboplatin. Figure S9. High-grade serous ovarian cancer Patient 259 derived CSC drive elevated CD206 expression in macrophages, and demonstrate a carboplatin resistant phenotype. Figure S10. Scatter plots for correlation of WNT5B with immune cell subsets in ovarian carcinoma. Table S1. List of primers used for qPCR experiments. (ZIP 1916 kb) [file 40425_2019_666_MOESM1_ESM.zip › SupFig2.tif]

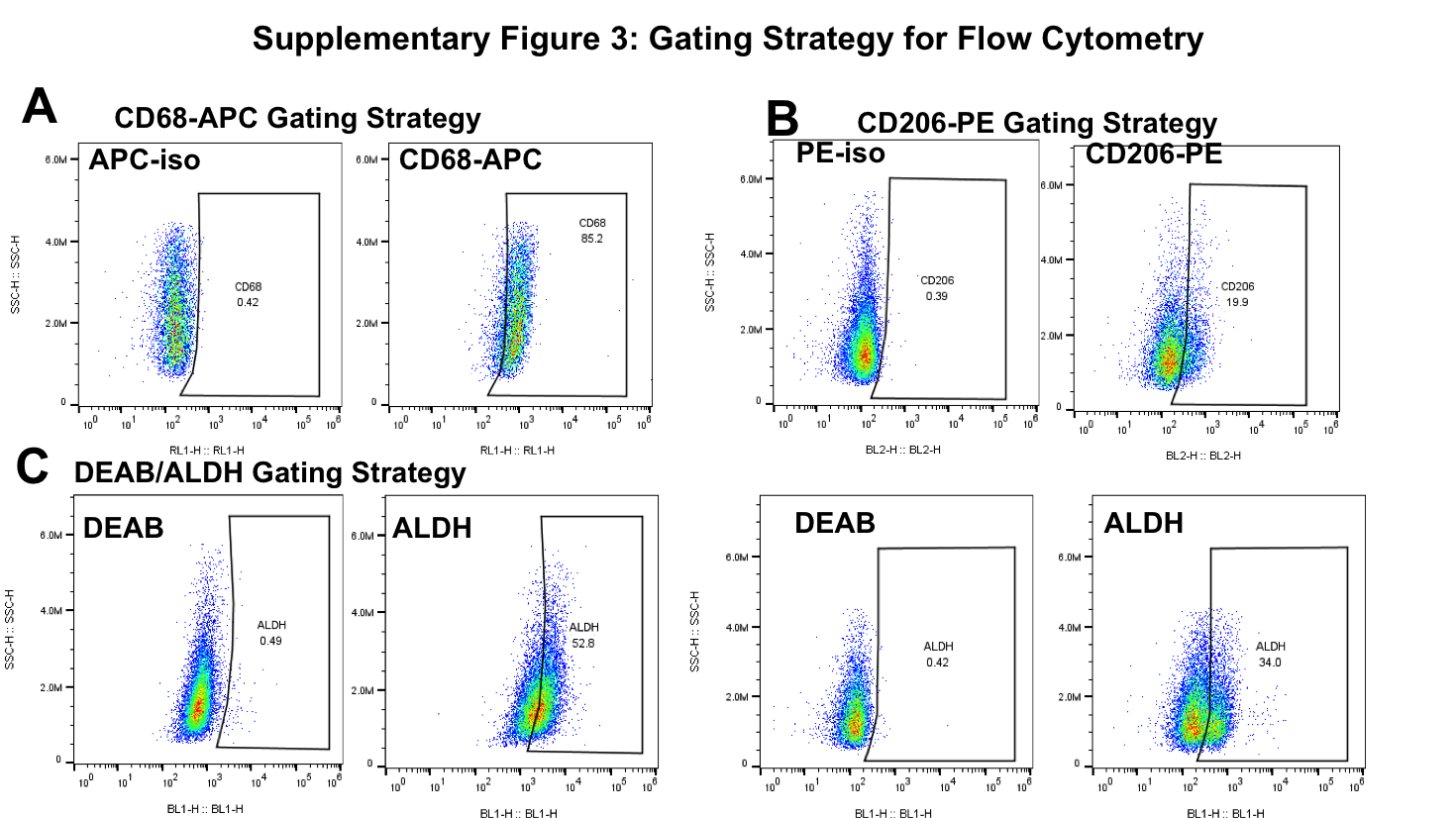

Supplement: Supplementary file 1 — Figure S1. U937 monocytes differentiated in 3D hanging drop arrays are equivalent to U937 monocytes differentiated in 2D. Figure S2. No change in proliferation in CSC compartments of hetero-spheroids. Figure S3. Gating strategy for Flow cytometry. Figure S4. Cancer cells do not significantly express the macrophage marker, CD206. Figure S5 CD163 expression is elevated in CSC/U937 M2 hetero-spheroids. Figure S6. Macrophages do not significantly express elevated ALDH. Figure S7. phospho-STAT3 is significantly reduced in CSC/shWNT5B-M2 hetero-spheroids compared to CSC/M2 hetero-spheroids. Figure S8. Kuramochi-CSC also drive elevated CD206 expression in macrophages, and polarized macrophages enrich ALDH+ cells in Kuramochi CSC and resistance to carboplatin. Figure S9. High-grade serous ovarian cancer Patient 259 derived CSC drive elevated CD206 expression in macrophages, and demonstrate a carboplatin resistant phenotype. Figure S10. Scatter plots for correlation of WNT5B with immune cell subsets in ovarian carcinoma. Table S1. List of primers used for qPCR experiments. (ZIP 1916 kb) [file 40425_2019_666_MOESM1_ESM.zip › SupFig3.tif]

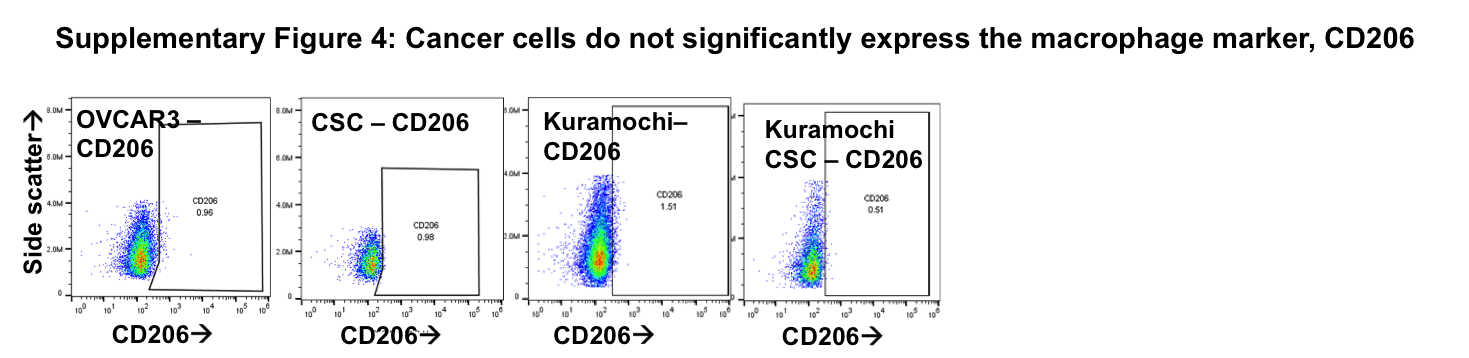

Supplement: Supplementary file 1 — Figure S1. U937 monocytes differentiated in 3D hanging drop arrays are equivalent to U937 monocytes differentiated in 2D. Figure S2. No change in proliferation in CSC compartments of hetero-spheroids. Figure S3. Gating strategy for Flow cytometry. Figure S4. Cancer cells do not significantly express the macrophage marker, CD206. Figure S5 CD163 expression is elevated in CSC/U937 M2 hetero-spheroids. Figure S6. Macrophages do not significantly express elevated ALDH. Figure S7. phospho-STAT3 is significantly reduced in CSC/shWNT5B-M2 hetero-spheroids compared to CSC/M2 hetero-spheroids. Figure S8. Kuramochi-CSC also drive elevated CD206 expression in macrophages, and polarized macrophages enrich ALDH+ cells in Kuramochi CSC and resistance to carboplatin. Figure S9. High-grade serous ovarian cancer Patient 259 derived CSC drive elevated CD206 expression in macrophages, and demonstrate a carboplatin resistant phenotype. Figure S10. Scatter plots for correlation of WNT5B with immune cell subsets in ovarian carcinoma. Table S1. List of primers used for qPCR experiments. (ZIP 1916 kb) [file 40425_2019_666_MOESM1_ESM.zip › SupFig4.tif]

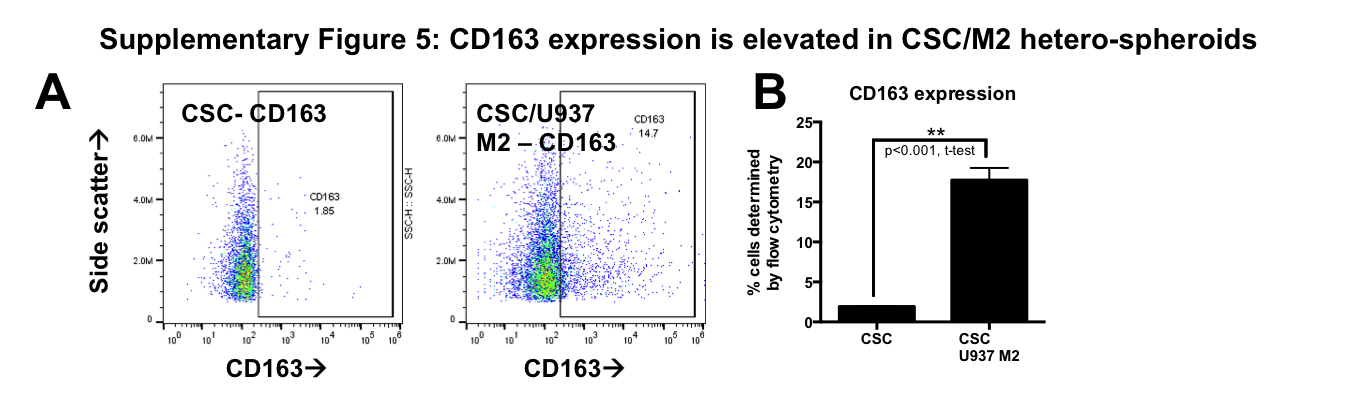

Supplement: Supplementary file 1 — Figure S1. U937 monocytes differentiated in 3D hanging drop arrays are equivalent to U937 monocytes differentiated in 2D. Figure S2. No change in proliferation in CSC compartments of hetero-spheroids. Figure S3. Gating strategy for Flow cytometry. Figure S4. Cancer cells do not significantly express the macrophage marker, CD206. Figure S5 CD163 expression is elevated in CSC/U937 M2 hetero-spheroids. Figure S6. Macrophages do not significantly express elevated ALDH. Figure S7. phospho-STAT3 is significantly reduced in CSC/shWNT5B-M2 hetero-spheroids compared to CSC/M2 hetero-spheroids. Figure S8. Kuramochi-CSC also drive elevated CD206 expression in macrophages, and polarized macrophages enrich ALDH+ cells in Kuramochi CSC and resistance to carboplatin. Figure S9. High-grade serous ovarian cancer Patient 259 derived CSC drive elevated CD206 expression in macrophages, and demonstrate a carboplatin resistant phenotype. Figure S10. Scatter plots for correlation of WNT5B with immune cell subsets in ovarian carcinoma. Table S1. List of primers used for qPCR experiments. (ZIP 1916 kb) [file 40425_2019_666_MOESM1_ESM.zip › SupFig5.tif]

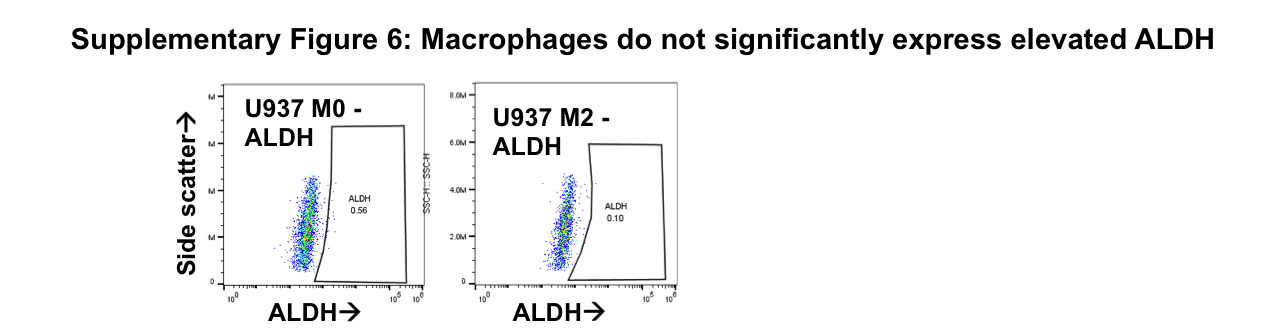

Supplement: Supplementary file 1 — Figure S1. U937 monocytes differentiated in 3D hanging drop arrays are equivalent to U937 monocytes differentiated in 2D. Figure S2. No change in proliferation in CSC compartments of hetero-spheroids. Figure S3. Gating strategy for Flow cytometry. Figure S4. Cancer cells do not significantly express the macrophage marker, CD206. Figure S5 CD163 expression is elevated in CSC/U937 M2 hetero-spheroids. Figure S6. Macrophages do not significantly express elevated ALDH. Figure S7. phospho-STAT3 is significantly reduced in CSC/shWNT5B-M2 hetero-spheroids compared to CSC/M2 hetero-spheroids. Figure S8. Kuramochi-CSC also drive elevated CD206 expression in macrophages, and polarized macrophages enrich ALDH+ cells in Kuramochi CSC and resistance to carboplatin. Figure S9. High-grade serous ovarian cancer Patient 259 derived CSC drive elevated CD206 expression in macrophages, and demonstrate a carboplatin resistant phenotype. Figure S10. Scatter plots for correlation of WNT5B with immune cell subsets in ovarian carcinoma. Table S1. List of primers used for qPCR experiments. (ZIP 1916 kb) [file 40425_2019_666_MOESM1_ESM.zip › SupFig6.tif]

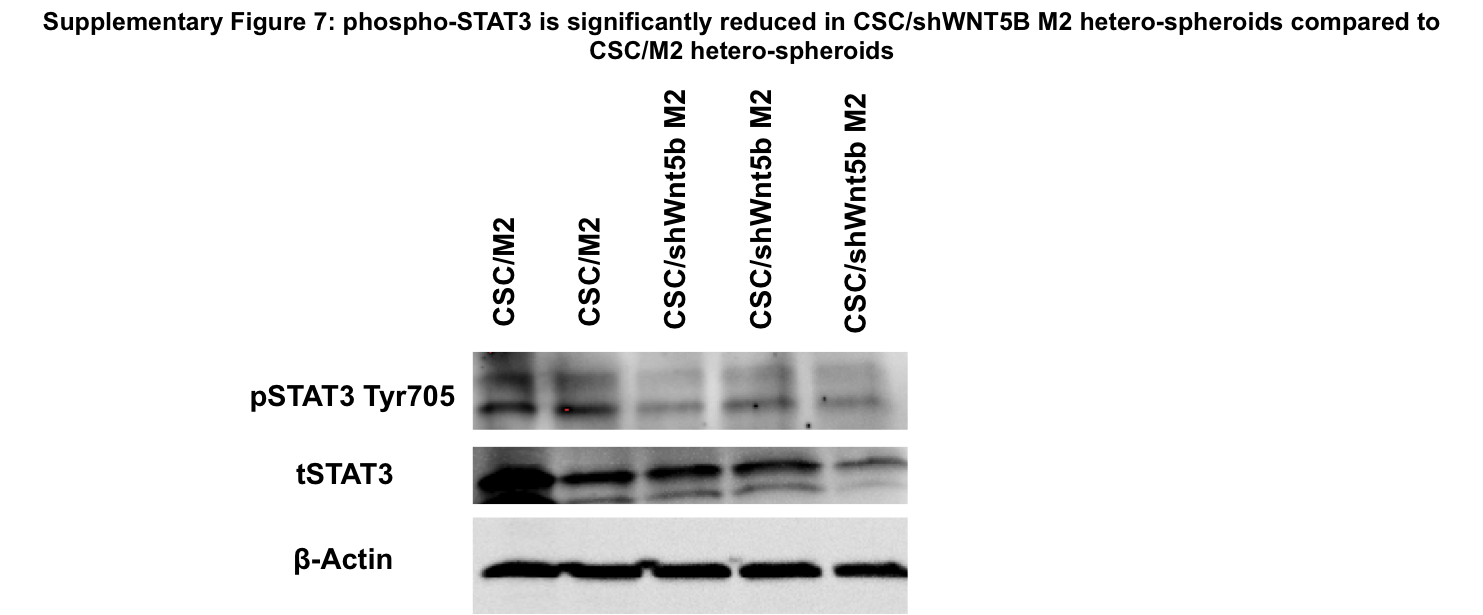

Supplement: Supplementary file 1 — Figure S1. U937 monocytes differentiated in 3D hanging drop arrays are equivalent to U937 monocytes differentiated in 2D. Figure S2. No change in proliferation in CSC compartments of hetero-spheroids. Figure S3. Gating strategy for Flow cytometry. Figure S4. Cancer cells do not significantly express the macrophage marker, CD206. Figure S5 CD163 expression is elevated in CSC/U937 M2 hetero-spheroids. Figure S6. Macrophages do not significantly express elevated ALDH. Figure S7. phospho-STAT3 is significantly reduced in CSC/shWNT5B-M2 hetero-spheroids compared to CSC/M2 hetero-spheroids. Figure S8. Kuramochi-CSC also drive elevated CD206 expression in macrophages, and polarized macrophages enrich ALDH+ cells in Kuramochi CSC and resistance to carboplatin. Figure S9. High-grade serous ovarian cancer Patient 259 derived CSC drive elevated CD206 expression in macrophages, and demonstrate a carboplatin resistant phenotype. Figure S10. Scatter plots for correlation of WNT5B with immune cell subsets in ovarian carcinoma. Table S1. List of primers used for qPCR experiments. (ZIP 1916 kb) [file 40425_2019_666_MOESM1_ESM.zip › SupFig7.tif]

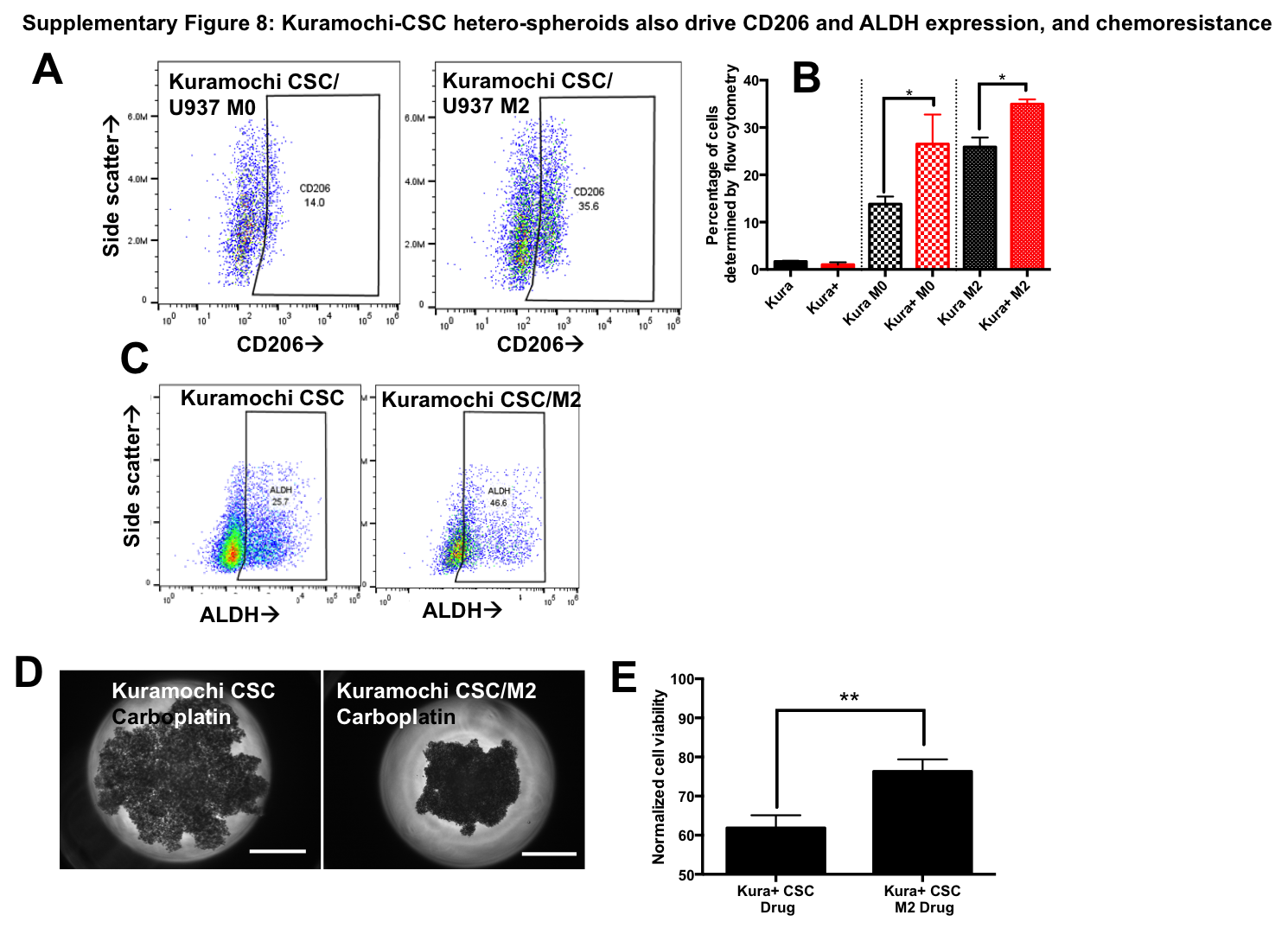

Supplement: Supplementary file 1 — Figure S1. U937 monocytes differentiated in 3D hanging drop arrays are equivalent to U937 monocytes differentiated in 2D. Figure S2. No change in proliferation in CSC compartments of hetero-spheroids. Figure S3. Gating strategy for Flow cytometry. Figure S4. Cancer cells do not significantly express the macrophage marker, CD206. Figure S5 CD163 expression is elevated in CSC/U937 M2 hetero-spheroids. Figure S6. Macrophages do not significantly express elevated ALDH. Figure S7. phospho-STAT3 is significantly reduced in CSC/shWNT5B-M2 hetero-spheroids compared to CSC/M2 hetero-spheroids. Figure S8. Kuramochi-CSC also drive elevated CD206 expression in macrophages, and polarized macrophages enrich ALDH+ cells in Kuramochi CSC and resistance to carboplatin. Figure S9. High-grade serous ovarian cancer Patient 259 derived CSC drive elevated CD206 expression in macrophages, and demonstrate a carboplatin resistant phenotype. Figure S10. Scatter plots for correlation of WNT5B with immune cell subsets in ovarian carcinoma. Table S1. List of primers used for qPCR experiments. (ZIP 1916 kb) [file 40425_2019_666_MOESM1_ESM.zip › SupFig8.tif]

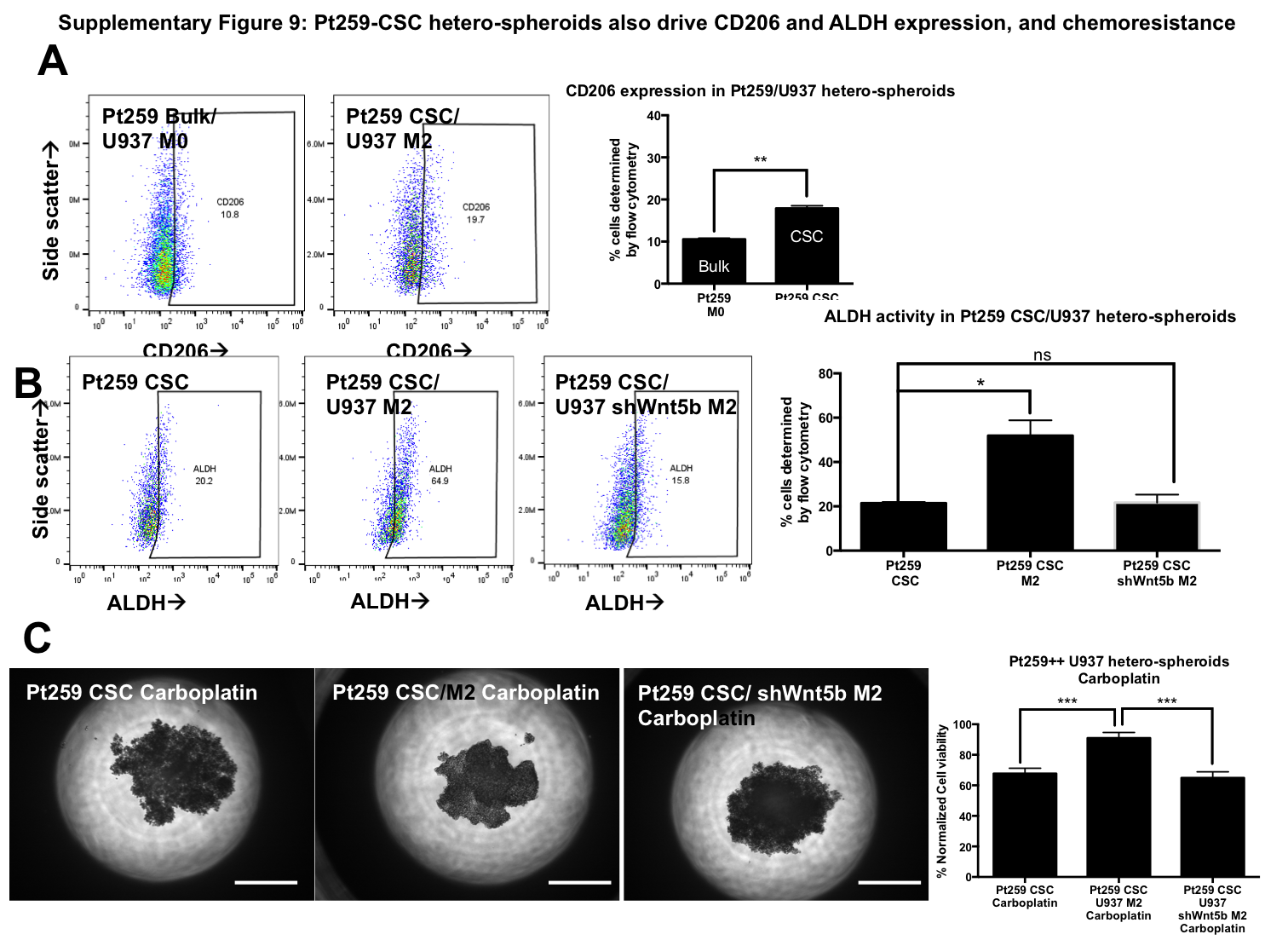

Supplement: Supplementary file 1 — Figure S1. U937 monocytes differentiated in 3D hanging drop arrays are equivalent to U937 monocytes differentiated in 2D. Figure S2. No change in proliferation in CSC compartments of hetero-spheroids. Figure S3. Gating strategy for Flow cytometry. Figure S4. Cancer cells do not significantly express the macrophage marker, CD206. Figure S5 CD163 expression is elevated in CSC/U937 M2 hetero-spheroids. Figure S6. Macrophages do not significantly express elevated ALDH. Figure S7. phospho-STAT3 is significantly reduced in CSC/shWNT5B-M2 hetero-spheroids compared to CSC/M2 hetero-spheroids. Figure S8. Kuramochi-CSC also drive elevated CD206 expression in macrophages, and polarized macrophages enrich ALDH+ cells in Kuramochi CSC and resistance to carboplatin. Figure S9. High-grade serous ovarian cancer Patient 259 derived CSC drive elevated CD206 expression in macrophages, and demonstrate a carboplatin resistant phenotype. Figure S10. Scatter plots for correlation of WNT5B with immune cell subsets in ovarian carcinoma. Table S1. List of primers used for qPCR experiments. (ZIP 1916 kb) [file 40425_2019_666_MOESM1_ESM.zip › SupFig9.tif]
